# Longitudinal tracking of subpopulation dynamics and molecular changes during LNCaP cell castration and identification of inhibitors that could target the PSA−/lo castration-resistant cells

**DOI:** 10.18632/oncotarget.7303

**Published:** 2016-02-10

**Authors:** Kiera Rycaj, Eun Jeong Cho, Xin Liu, Hsueh-Ping Chao, Bigang Liu, Qiuhui Li, Ashwini K. Devkota, Dingxiao Zhang, Xin Chen, John Moore, Kevin N. Dalby, Dean G. Tang

**Affiliations:** ^1^ Department of Epigenetics and Molecular Carcinogenesis, University of Texas MD Anderson Cancer Center, Science Park, Smithville, TX 78957, USA; ^2^ College of Pharmacy, Targeted Therapeutic Drug Discovery and Development Core Facility (TTDDD), The University of Texas at Austin, Austin, TX 78712, USA; ^3^ Cancer Stem Cell Institute, Research Center for Translational Medicine, East Hospital, Tongji University School of Medicine, Shanghai 200120, China; ^4^ Centers for Cancer Epigenetics, Stem Cell and Developmental Biology, RNA Interference and Non-coding RNAs, and Molecular Carcinogenesis, University of Texas MD Anderson Cancer Center, Houston, TX 77030, USA

**Keywords:** prostate cancer, castration resistance, cancer stem cells, cellular heterogeneity, differentiation

## Abstract

We have recently demonstrated that the undifferentiated PSA^−/lo^ prostate cancer (PCa) cell population harbors self-renewing long-term tumor-propagating cells that are refractory to castration, thus representing a therapeutic target. Our goals here are, by using the same lineage-tracing reporter system, to track the dynamic changes of PSA^−/lo^ and PSA^+^ cells upon castration *in vitro*, investigate the molecular changes accompanying persistent castration, and develop large numbers of PSA^−/lo^ PCa cells for drug screening. To these ends, we treated LNCaP cells infected with the PSAP-GFP reporter with three regimens of castration, i.e., CDSS, CDSS plus bicalutamide, and MDV3100 continuously for up to ~21 months. We observed that in the first ~7 months, castration led to time-dependent increases in PSA^−/lo^ cells, loss of AR and PSA expression, increased expression of cancer stem cell markers, and many other molecular changes. Meanwhile, castrated LNCaP cells became resistant to high concentrations of MDV3100, chemotherapeutic drugs, and other agents. However, targeted and medium-throughput library screening identified several kinase (e.g., IGF-1R, AKT, PI3K/mTOR, Syk, GSK3) inhibitors as well as the BCL2 inhibitor that could effectively sensitize the LNCaP-CRPC cells to killing. Of interest, LNCaP cells castrated for >7 months showed evidence of cyclic changes in AR and the mTOR/AKT signaling pathways potentially involving epigenetic mechanisms. These observations indicate that castration elicits numerous molecular changes and leads to enrichment of PSA^−/lo^ PCa cells. The ability to generate large numbers of PSA^−/lo^ PCa cells should allow future high-throughput screening to identify novel therapeutics that specifically target this population.

## INTRODUCTION

Cellular heterogeneity represents a fundamental challenge to cancer treatment. Human tumors manifest significant histo-structural and cellular heterogeneity containing phenotypically differentiated and undifferentiated subpopulations of tumor cells. Prostate cancer (PCa) is no exception: well-differentiated, low-grade tumors (i.e., Gleason 6-7) contain glandular structures with tumor cells expressing differentiation markers such as androgen receptor (AR) and prostate-specific antigen (PSA). In contrast, poorly differentiated PCa lacks glandular structures and differentiated cells. Androgen signaling has been the primary target for PCa treatment for >70 years. However, androgen deprivation therapy (ADT) only achieves short-term efficacy due to the emergence of castration-resistant disease (CRPC). This may be partly related to the cellular heterogeneity of prostate tumors, in which distinct subpopulations of drug-resistant tumor cells can survive ADT and then repopulate the tumor [1]. Recent evidence suggests that the population of PCa cells in untreated primary tumors that survive ADT may expresses little/no AR (i.e., AR^−/lo^) and possesses many cancer stem cell (CSC) properties [2–4]. Supporting this concept of prostate CSCs (PCSCs) is the ample literature that indicates that untreated patient tumors contain a fraction of AR^−/lo^ PCa cells that tend to increase as tumors progress and especially upon treatment and recurrence [2, 3, 5, 6].

Untreated PCa also harbors PCa cells that lack significant expression of PSA [2, 3], a direct target of AR. These PSA^−/lo^ PCa cells, like AR^−/lo^ cells, also tend to increase in advanced and recurrent PCa [2, 3]. Furthermore, AR and PSA protein expression in PCa is frequently discordant with some PCa cells completely lacking the expression of either or both [2, 3, 5] Using a lentiviral PSA reporter (i.e., PSAP-GFP), we were able to separate the PSA^−/lo^ PCa cells from the PSA^+^ counterparts to study their distinct biological and tumorigenic properties. We find that the PSA^−/lo^ PCa cells are quiescent, refractory to stresses including androgen deprivation, exhibit high clonogenic potential, possess long-term tumor-propagating capacity, preferentially express stem cell genes, and can undergo asymmetric division to generate differentiated cells. Important, the PSA^−/lo^ PCa cell population is enriched in AR^−/lo^ cells, resist castration treatment, and can initiate robust tumor development in fully castrated mice [2, 3].

The ultimate goal of our research is to better characterize and find novel therapeutics targeting the PSA^−/lo^ PCSC pool [2, 3]. The PSA^−/lo^ population in untreated PCa cell cultures and reporter tumors is generally rare being < 1-20% [2, 3]. Also, when these cells are freshly purified via flow cytometry, they are fragile and not amenable to drug screening. To overcome these limitations and to dynamically track the changes in PSA^−/lo^ and PSA^+^ cell subpopulations in response to androgen deprivation, here we chronically exposed LNCaP cells to 3 regimens of castration *in vitro*, to mimic continuous treatment of patient prostate tumors by anti-androgens. We report that persistent castration of LNCaP cells leads to, within the first ~7 months, the generation of homogenous AR^−/lo^ PSA^−/lo^ cell population that has undergone many molecular changes, in addition to alterations in the AR pathway, and is highly refractory to MDV3100 and other therapeutic drugs but amenable for high throughput drug screening and remains sensitive to BCL2 antagonist and several kinase inhibitors.

## RESULTS

### Establishment of traceable LNCaP-CRPC cells

**Figure 1:**
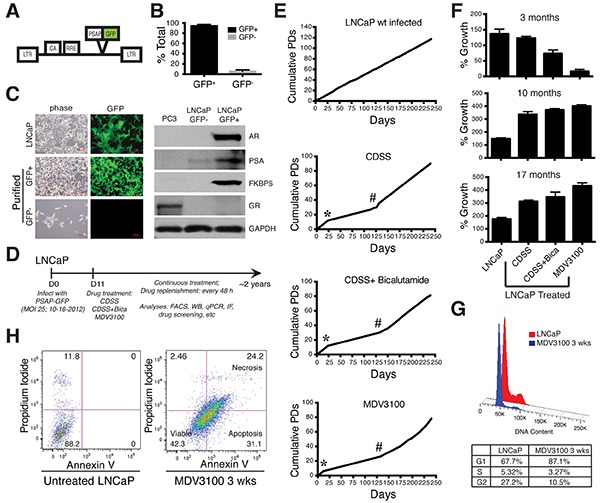
Establishment of long-term LNCaP-CRPC cells and their overall growth kinetics **A.** The PSAP-GFP lentivector, in which the GFP reporter was driven by a PSA promoter (PSAP). LTR, long-terminal repeat; GA. *gag* gene; RRE, Rev responsive element. **B.** Quantification of GFP^+^ (PSA^+^) and GFP^−/lo^ (PSA^−/lo^) cells in freshly infected, untreated LNCaP cells (n=12). **C.** Freshly purified GFP^−/lo^ LNCaP cells express little AR or its targets PSA and FKBP5. Shown on the left are representative images of bulk PSA-GFP infected (72 h) LNCaP cells (top panels), and FACS-purified GFP^−^ and GFP^+^ cells cultured overnight (lower panels). Original magnifications, ×100. Shown on the right are WB (Western blot) gel images of the molecules indicated in freshly sorted GFP^+/−^ LNCaP cells. **D.** Timeline for establishing LNCaP-CRPC sublines. FACS, fluorescence-activated cell sorting; WB, western blot; IF, immunofluorescence staining. **E.** Population doublings (PDs) of LNCaP-GFP (parental) and LNCaP-CRPC cells for up to ~250 days. Cumulative PDs were calculated using the equation: PD = (Nf/Ni)/2, where Nf is the final cell count, and Ni is the initial cell count. Asterisks indicate the “crisis” periods (~2-3 weeks) when there were little net PD increases. The # symbols indicate the time (~4 months) when the LNCaP-CRPC cultures started aggressive growth patterns. **F.** Different growth kinetics of LNCaP-CRPC cells at 3, 10, or 17 months in comparison to LNCaP-GFP cells. The 4 types of LNCaP cells were plated, in quadruplicate, in 12-well plates (5,000 cells/well) and viable cells were quantified using Trypan blue 10 days post plating. **G.** MDV3100 induces cell-cycle arrest in LNCaP cells. Histogram plots presenting total DNA content quantification in cells after 3 weeks (wks) of MDV3100 (10 μM) treatment compared to untreated parental LNCaP cells (top). A table below displays cell percentages in G_1_, S and G_2_/M phases. **H.** MDV3100 induces cell death in LNCaP cells. FACS dot plots displaying percentages of viable, apoptotic, and necrotic cell populations after 3 weeks of MDV3100 (10 μM) treatment compared to parental LNCaP cells.

LNCaP, initially established from a lymph node metastasis [7, 8], is the most commonly used PCa cell line and has been widely utilized to derive sublines and clonal cultures to model PCa cell response to androgen deprivation (e.g., [9-22]). All these previous studies used bulk LNCaP cells without taking into account the subpopulation heterogeneity [2, 3]. For example, regular LNCaP cultures have cells expressing high levels of AR (i.e., AR^+^) and PSA (i.e., PSA^+^) as well as cells expressing little/no AR (i.e., AR^−/lo^) and PSA (PSA^−/lo^) [2, 3; also see Supplementary Figure S2, below]. We have recently employed a lentiviral-based reporter system in which a PSA promoter (PSAP) drives GFP or RFP (Figure 1A; Supplementary Figure S1A) to PROSPECTIVELY separate the two lineage-related subpopulations (i.e., PSA^+^ and PSA^−/lo^) of PCa cells [2]. Comprehensive studies demonstrate that the phenotypically undifferentiated PSA^−/lo^ PCa cell population harbors self-renewing long-term tumor-propagating cells that are intrinsically refractory to castration [2, 3]. In this project, we first aim to employ this tracing system to longitudinally track the dynamic changes of the same two subpopulations of LNCaP cells in response to chronic androgen deprivation *in vitro*.

LNCaP cells regularly cultured in 7% FBS-containing medium and infected with the PSAP-GFP lentiviral reporter (Figure 1A) contained 5.39 ± 3.18% (*n* = 12) GFP^−/lo^ cells (i.e., bottom 6-10% GFP^−/lo^ population on FACS) (Figure 1B). Freshly purified GFP^−/lo^ LNCaP cells expressed little AR or its targets PSA and FKBP5, analogous to the AR^−^ PSA^−^ PC3 cells (Figure 1C). In contrast, the corresponding GFP^+^ cells (i.e., top 5-10% of GFP-bright cells on FACS) expressed all three proteins (Figure 1C). Neither cell population expressed glucocorticoid receptor (GR) (Figure 1C), which was recently reported to confer resistance to antiandrogens [23]. As the PSAP-GFP lentiviral system faithfully reports endogenous PSA expression [2], in foregoing experiments, we often interchangeably use GFP^+^/GFP^−/lo^ and PSA^+^/PSA^−/lo^.

**Figure 2:**
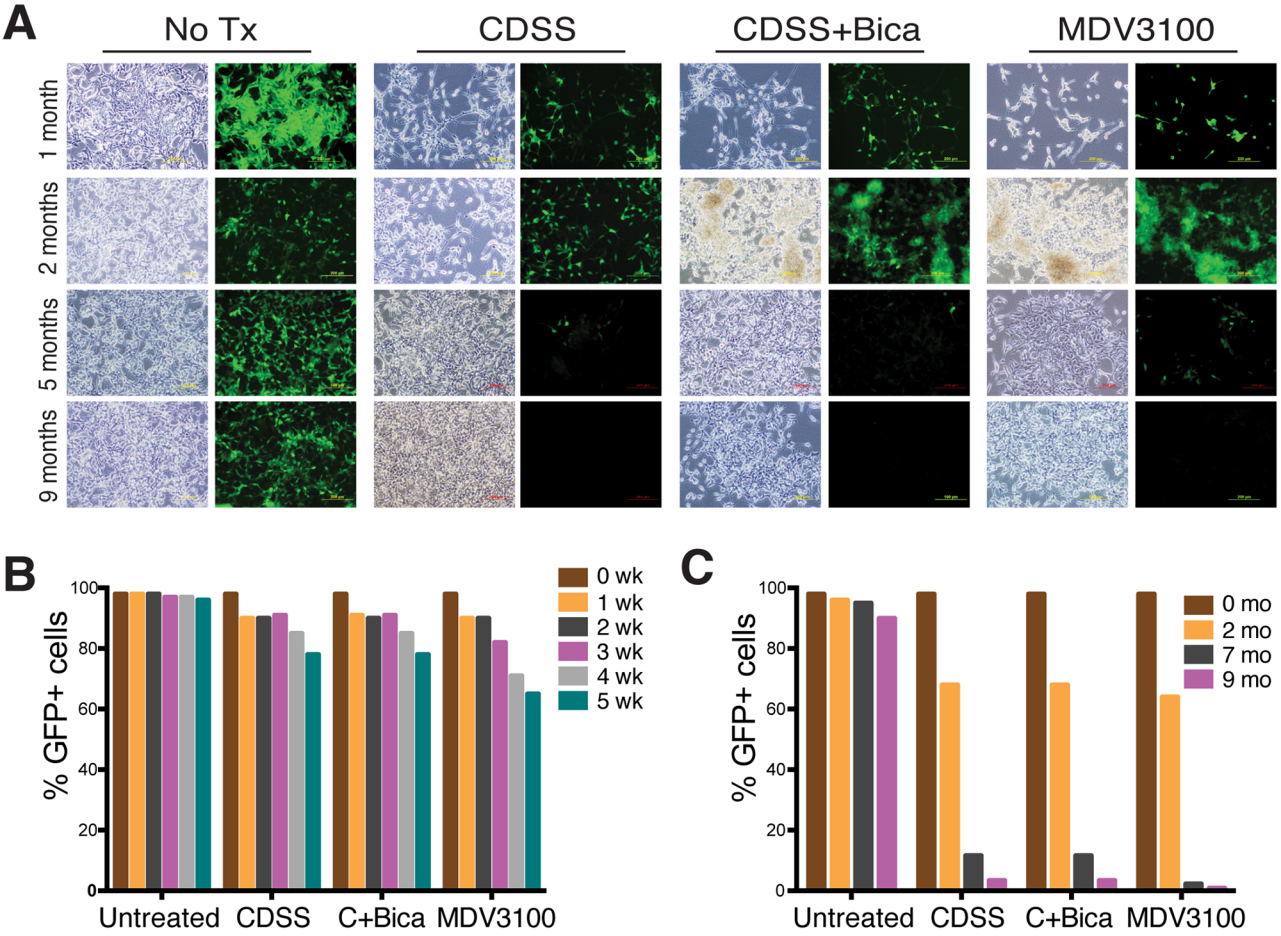
Time-dependent decrease in PSA^+^ cells in response to castration **A.** Representative phase and GFP images of LNCaP and 3 types of LNCaP-CRPC cells treated for 1, 2, 5, and 9 months. **B.** Quantification of GFP^+^ percentage in short-term treated LNCaP cells. **C.** Quantification of GFP^+^ percentage in long-term treated LNCaP cells.

We infected LNCaP cells with the PSAP-GFP at a multiplicity of infection (MOI) of 25, at which virtually all cells were infected (Figure 1C; 2). We then treated the infected LNCaP cells with 3 regimens of castration: charcoal dextran-stripped serum (CDSS), CDSS with bicalutamide (10 μM), and MDV3100 (Enzalutamide, 10 μM) continuously for up to ~2 years (Figure 1D), which resulted in the long-term castration-resistant LNCaP sublines that we termed LNCaP-CRPC cells, i.e., LNCaP-CDSS, LNCaP-CDSS+Bicalutamide, and LNCaP-MDV. We first characterized the overall growth kinetics of the LNCaP-CRPC sublines (Figure 1E–1F). As shown in Figure 1E, infected but untreated LNCaP-GFP (parental) cells exhibited steady increases in cumulative population doublings (PDs). The 3 treated LNCaP cell types all grew slower in the beginning and hit a “bump” or “crisis” point around 2-3 weeks when there was little net increase in PDs (Figure 1E; asterisks). Then the treated cells began to grow with a steady increase in PDs, although at slower paces than the untreated LNCaP-GFP cells (Figure 1E). Indeed, after 3 months of treatment, all three LNCaP-CRPC lines showed much lower end-point live cell numbers (Figure 1F, top), suggesting that they were less proliferative and/or more susceptible to cell death. Interestingly, at ~4 months (125 days), there was a noticeable increase in the growth kinetics in all 3 LNCaP-CRPC sublines (Figure 1E). In support, all 3 LNCaP-CRPC cultures continuously treated for 10 or 17 months showed significantly more live cell numbers compared to the time-matched control LNCaP-GFP cells (Figure 1F).

We further characterized LNCaP-GFP and LNCaP-MDV cells at crisis point (3 weeks) and found that MDV3100 treatment led to both increased cell-cycle arrest (Figure 1G) and cell death (Figure 1H). Specifically, more LNCaP-MDV cells remained in the G1 phase compared to LNCaP-GFP cells (87.1% versus 67.7%) and less were at the G2/M phase (27% versus 10.5%) (Figure 1G). With respect to cell death, ~90% untreated LNCaP-GFP cells were viable; in contrast, only 42% LNCaP-MDV cells treated for 3 weeks were viable with 31% of cells being apoptotic and 24% being necrotic (Figure 1H).

### Castrated LNCaP cultures gradually lose PSA^+^ cells as well as AR and PSA expression

We monitored dynamic changes in GFP^+^ cells in chronically castrated LNCaP cells (Figure 2). As early as 1 week after treatment, there was a ~5-10% decrease in GFP^+^ population in all 3 conditions (Figure 2B). By 4-5 weeks, there were 15-25% decreases in GFP^+^ cells with concomitant increases in GFP^−/lo^ (PSA^−/lo^) cells, with MDV3100 showing the strongest effect (Figure 2A–2B). By 2 months, the GFP^+^ population dropped to ~70% in all 3 LNCaP-CRPC cultures and at 5-7 months the GFP^+^ cell population dramatically decreased (Figure 2A and 2C). By 9 months after treatment, there were barely detectable GFP^+^ cells in the 3 LNCaP-CRPC sublines (Figure 2A and 2C). In contrast to these castration-induced changes, untreated LNCaP-GFP cells remained mostly GFP^+^ over the 9-month treatment period (Figure 2).

We further tracked the changes in PSA^+^ cells in castrated LNCaP cells infected with a tracing vector that contained a built-in RFP reporter driven by CMV promoter (Supplementary Figure S1; S2). This dual reporter system allowed us to monitor alterations of PSA^+^ (GFP^+^) cells in infected (i.e., RFP^+^) cells (Supplementary Figure S1A). As observed with the PSAP-GFP reporter, all three castration regimens led to a gradual decrease in GFP^+^ cells whereas all surviving cells remained RFP^+^ (Supplementary Figure S1B-S1D). By 6 months, only very few LNCaP-CRPC cells were dimly GFP^+^ (Supplementary Figure S1C).

**Figure 3:**
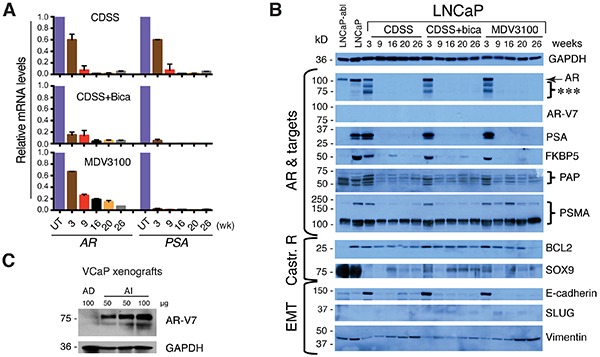
Loss of AR and PSA expression in LNCaP-CRPC cell lines **A.** *AR* and *PSA* mRNA expression determined by qPCR in LNCaP-CRPC cells. **B.** Immunoblotting of AR and other molecules indicated in LNCaP, LNCaP-abl, and LNCaP-CRPC cells treated for 3-26 weeks. ***indicates low-M.W. AR species. **C.** Immunoblotting of AR-V7 using an AR-V7 specific antibody in an androgen-dependent (AD) VCaP xenograft (grown in intact NOD/SCID/γ mice) and an androgen-independent (AI) VCaP xenograft (grown in castrated NOD/SCID/γ mice). Protein amount loaded per lane was indicated.

Accompanying the loss of PSA^+^ cells was a time-dependent decrease in *AR* and *PSA* mRNAs in the three LNCaP-CRPC cultures (Figure 3A). Western blotting showed that at 3 weeks of treatment, LNCaP-CRPC cells expressed AR protein and its 4 targets, i.e., PSA, FKBP5, PAP, and PSMA (Figure 3B). Interestingly, the 3-week treated cultures showed several lower AR bands that could represent the AR splice variants though none of these bands represented AR-V7 since an AR-V7 specific antibody (Figure 3C) failed to detect any products in the LNCaP-CRPC cultures (Figure 3B). Remarkably, at 9-26 weeks, all 3 LNCaP-CRPC lines lost AR and PSA protein expression and also showed decreased expression of other AR targets (Figure 3B). In the independent experiment using the dual reporter system, AR and PSA proteins decreased as early as 1 week after the initiation of castration (Supplementary Figure S1E). Interestingly, the LNCaP-abl [9], a castration-resistant LNCaP subline commonly used to study resistance mechanisms (24), were AR^+^ PSA^−^ (Figure 3B), as we reported recently [3].

Together, these longitudinal tracking experiments indicate that *in vitro* castration leads to a loss of differentiated AR^+^ PSA^+^ LNCaP cells, as further supported by immunofluorescence staining of AR and PSA in the 5-month (5-mo) LNCaP-CRPC cells (Supplementary Figure S2).

### LNCaP-CRPC cells also demonstrate changes in many other signaling molecules

In addition to the AR signaling pathway, we also examined changes in several molecules related to castration resistance (BCL2 and SOX9), epithelial-mesenchymal transition or EMT (E-cadherin, SLUG, and vimentin), and CSCs (i.e., CD44, integrin α2β1, and ABCG2) [2-4, 20, 25-33] (Figure 3B; Supplementary Figure S3). Flow cytometry revealed time-dependent increases in cells expressing high levels of 3 CSC markers in LNCaP-CRPC cultures (Supplementary Figure S3), consistent with the notion that castration enriches for stem-like cancer cells (2-4). All other molecules showed variegated changes (Figure 3B). For example, BCL2, an anti-apoptotic molecule shown previously to be upregulated during castration [20], actually exhibited slight decreases in our LNCaP-CRPC cells (Figure 3B). SOX9, a stem cell molecule recently reported to be regulated by AR [25], showed rapid downregulation in 3-week castrated LNCaP cells and then slightly increased in long-term LNCaP-CRPC cells (Figure 3B). Castration has been linked to EMT [31–33]. However, our LNCaP-CRPC cells showed decreased E-cadherin and subtle changes in SLUG and vimentin (Figure 3B). N-cadherin was not expressed in parental LNCaP cells nor was it induced in LNCaP-CRPC cells (data not shown). These results suggest that castration resistance in LNCaP cells in our experimental conditions is not associated with apparent EMT.

### Single cell tracking of LNCaP cell response to castration reveals distinct drug sensitivity in parental LNCaP vs. LNCaP-MDV cells

**Figure 4:**
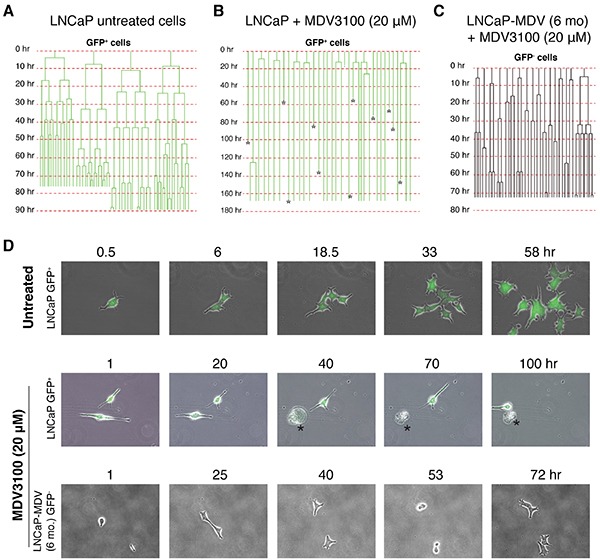
Time-lapse tracking of LNCaP cells in response to MDV3100 **A.** Cell division mode and cell-cycle transit times in untreated GFP^+^ LNCaP cells in regular serum-containing medium as determined by time-lapse videomicroscopy. Shown are 4 representative GFP^+^ cells that underwent rapid symmetrical cell divisions. Time scale is shown for each cell recorded. **B.** Cell division mode and cell-cycle times in GFP^+^ LNCaP cells acutely treated with MDV3100 (10 μM). Asterisks indicate dead cells. **C.** Cell division mode and cell-cycle transit times in LNCaP-MDV (6-mo) cells treated with MDV3100 (10 μM). Note that the 6-month LNCaP-MDV cells were all GFP^−^. **D.** Representative time-lapse images for data presented in A-C.

We employed time-lapse video microscopy [2, 3] to further monitor the survival and division mode of single parental LNCaP (i.e., LNCaP-GFP) and LNCaP-MDV (both at 6-mo) cells to fresh MDV3100 treatment (Figure 4). As we observed previously [2, 3], in untreated LNCaP cultures, GFP^+^ (PSA^+^) cells, which represented the bulk (i.e., ~95%), underwent rapid symmetrical cell divisions with average cell-cycle transit time of 16.5±8.4 h (n=48 cells) (Figure 4A; Figure 4D, top). In response to acute MDV3100 treatment (10 μM), most GFP^+^ LNCaP cells underwent cell-cycle arrest and/or death without cell division (Figure 4B; Figure 4D, middle). Many GFP^+^ LNCaP cells never divided during the entire >160 h recording period (Figure 4B). In contrast to MDV3100-treated parental GFP^+^ LNCaP cells, the 6-mo LNCaP-MDV cells, all of which were GFP^−^, behaved like the untreated GFP^+^ parent LNCaP cells and divided fast in the presence of MDV3100 with a cell-cycle time of 25.6±16.7 h (n=35 cells) (Figure 4C; Figure 4D, bottom panels). This defined single-cell tracking experiment provides direct evidence that 1) acute MDV3100 treatment of PSA^+^ LNCaP cells causes prominent cell-cycle arrest and/or cell death, and 2) the chronically castrated PSA^−/lo^ LNCaP-CRPC cells, unlike the parental PSA^+^ cells, no longer respond to MDV3100.

### LNCaP-CRPC cells are refractory to further castration and chemotherapeutic drugs but sensitive to anti-BCL-2 and several kinase inhibitors

**Figure 5:**
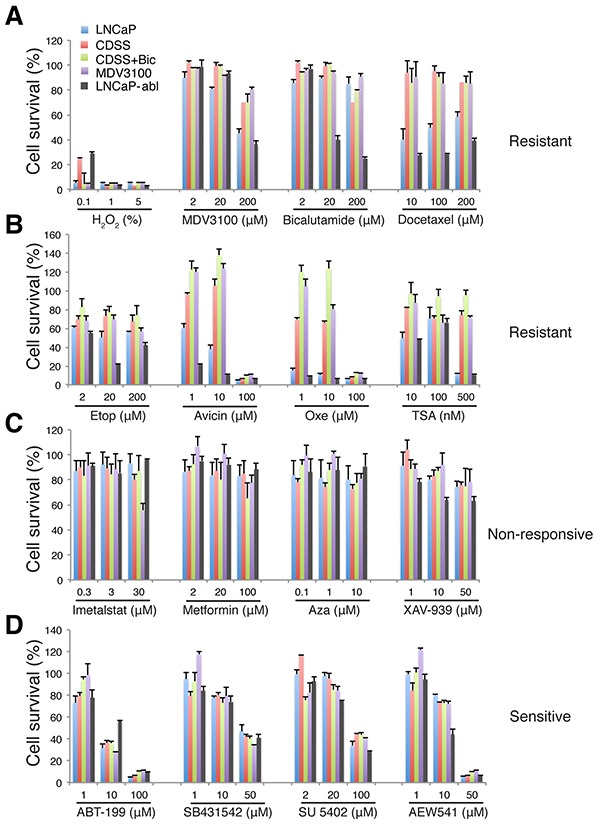
Candidate library screening in LNCaP-CRPC cells Presented is the relative cell survival (%), as determined by AlmarBlue assay, in LNCaP-GFP (LNCaP; 5-mo), LNCaP-CRPC (5-mo), and LNCaP-abl cells when exposed to 15 compounds in the candidate library for 72 h. Compound concentrations were selected based on the reported IC_50_ values (Supplementary Table S2). The response patterns were classified into 4 categories.

To test whether the long-term LNCaP-CRPC cells would be refractory to ‘therapeutic’ treatments other than antiandrogens, we treated 5-mo LNCaP-GFP and the 3 LNCaP-CRPC cell types, for 72 h, with a ‘candidate library’ of 15 compounds that included: 2 antiandrogens (MDV3100 and Bicalutamide), 2 chemotherapeutic drugs (docetaxel and etoposide), 2 plant-derived experimental drugs (Avicin and Oxetane; [34]), a telomerase inhibitor (Imetalstat, also called GRN163L; [35]), Metformin (an antidiabetic drug shown to inhibit CSCs; [36]), a selective BCL-2 inhibitor ABT-199 [37, 38], 2 epigenetic inhibitors, i.e., 5-Aza-2′-deoxycytidine (Aza, an inhibitor of DNA methyltransferase) and trichostatin A (TSA, a histone deacetylase inhibitor), and 4 inhibitors of signaling pathways, i.e., XAV-939 that inhibits Wnt/b-catenin; SB431542 that inhibits TGFBR1; SU 5402 that inhibits VEGFR1 and FGFR1; and AEW541 that inhibits the IGF-1R (Figure 5; Supplementary Table S2). We used H_2_O_2_ as a control, which non-selectively killed all cell types at ≥5 μM, and also compared with the drug sensitivities of LNCaP-abl cells (Figure 5).

This ‘targeted’ drug screening revealed that the 5-mo LNCaP-CRPC cells that lacked AR and PSA expression, within 72 h, were resistant to antiandrogens, chemotherapeutic drugs (docetaxel, etoposide, Avicin, and Oxetane), and TSA (Figure 5A–5B), non-responsive to Imetastat, Metformin, Aza, and XAV-939 (Figure 5C) but sensitive to ABT-199, SB431512, SU 5402, and AEW541 (Figure 5D). Specifically, MDV3100 dose-dependently inhibited LNCaP-GFP cells but the LNCaP-CRPC cells showed resistance to even 200 μM of MDV3100 (Figure 5A). Bicalutamide, known to be less potent that MDV3100 in antagonizing AR (Supplementary Table S2), did not affect LNCaP-GFP or LNCaP-CRPC cells, even at 200 μM. Interestingly, LNCaP-CRPC cells demonstrated most prominent resistance to docetaxel, Avicin, Oxetane, and TSA compared to LNCaP-GFP cells (Figure 5A–5B). On the other hand, both LNCaP-GFP and LNCaP-CRPC cells did not appreciably respond to Imetalstat, metformin and Aza and both showed only slight responses to XAV-939 (Figure 5C), suggesting that either these inhibitors were ineffective or needed >72 h to manifest effects. Importantly, however, the 5-mo LNCaP-CRPC cells, like LNCaP-GFP cells, responded, in a dose-dependent manner, to ABT-199 and 3 kinase inhibitors, i.e., SB431542, SU 5402, and AEW541 (Figure 5D), implicating potentially critical roles of BCL2 and TGFBR1, VEGFR1/FGFR1, and IGF-1R signaling in the survival of LNCaP-CRPC cells.

**Figure 6:**
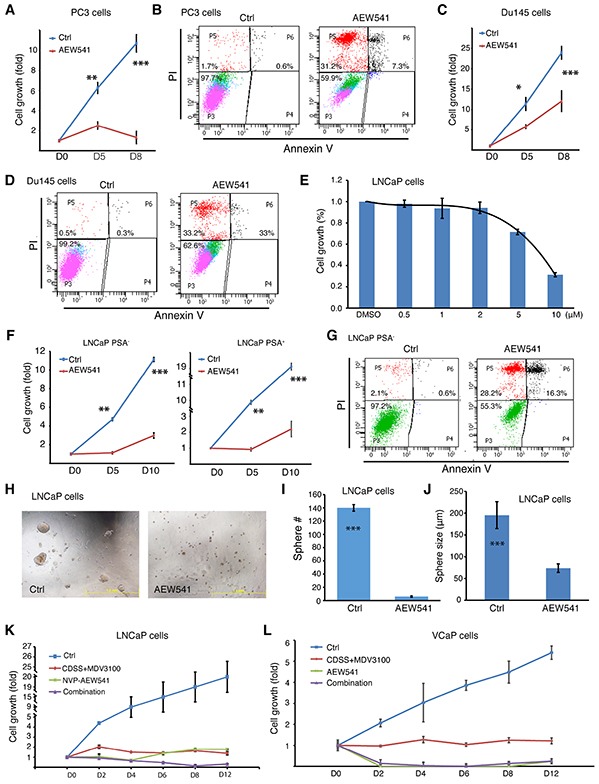
Effects of IGF-1R inhibitor AEW541 on AR^−^/PSA^−^ PC3 and Du145 cells and PSA^−/lo^ LNCaP cells **A-D.** PC3 and Du145 cells are sensitive to AEW541. In A and C, PC3 (A) or Du145 (C) cells (2,000/well) were plated in 96-well plates. After cells attached to the plate (12 h), vehicle control (Ctrl; DMSO) or AEW541 (10 μM final concentration) was added to the wells and relative live cell number was determined by WST-1 assays at day 0, 5, and 8 (see Supplementary Methods). Presented are fold changes in cell numbers (**P<0.05; ***P<0.001). Apoptosis assays were performed using the Vybrant kit and shown in B and D are representative histograms showing AEW541-induced cell death. **E.** Dose response of AEW541 in LNCaP cells, as measured by WST-1 assays. **F-G.** The PSA^−/lo^ LNCaP cells are susceptible to AEW541. Purified PSA^+^/PSA^−/lo^ LNCaP cells were utilized in WST-1 cell growth assays (F) and Vybrant apoptosis assays (G) in the presence of DMSO (Ctrl) or AEW541 (10 μM). **P<0.05; ***P<0.001. **H-J.** AEW541 inhibits LNCaP sphere formation. Bulk LNCaP cells were plated at the clonal level in methylcellulose based sphere assays in the presence of DMSO (Ctrl) or AEW541 (10 μM). 2 weeks later, sphere number and size were measured (***P<0.001). Shown in H are representative images. **K-L.** AEW541 is more effective than CDSS+MDV3100 in killing LNCaP and VCaP (E) cells. Bulk LNCaP or VCaP cells were plated at 2,000 cells/well in triplicate in 96-well plates and cultured in RPMI-7% FBS. Cell number in each well was assessed by WST-1 assays in the presence of DMSO (Ctrl). Cells were then treated with DMSO (ctrl), CDSS+MDV3100 (20 μM), AEW541 (10 μM), or combination. Live cells were determined at the indicated time points, and results are presented as fold increase. In K, the difference between Ctrl vs. other conditions at D2-D12 was all statistically significant (P<0.01). In L, the difference between Ctrl vs. the other three conditions at D2-D12 was statistically significant (P<0.001). The difference between CDSS+MDV3100 vs. AEW541 or combination at D2-D12 was also statistically significant (P<0.05).

The results with AEW541 were especially interesting as we previously showed the involvement of IGF-1R in positively regulating the PSA^−/lo^ LNCaP cells [2] and the IGF/IGF-1R signaling pathway has been implicated in CRPC progression [39]. Indeed, AEW541 prominently inhibited growth of AR^−^ PSA^−^ Du145 and PC3 cells by causing apoptosis (Figure 6A–6D). AEW541 also inhibited the bulk LNCaP cells in a dose-dependent manner (Figure 6E). Significantly, although the PSA^−/lo^ PCa cells are highly resistant to castration, prooxidants, and some chemotherapeutic drugs [2, 3], AEW541 exhibited inhibitory effects on both PSA^+^ as well as PSA^−/lo^ LNCaP cells by causing increased apoptosis (Figure 6F–6G). AEW541 also inhibited the spherogenic activities in LNCaP cells (Figure 6H–6J). Finally, we found that AEW541 at 10 μM was as effective as CDSS+MDV3100 (20 μM) in suppressing LNCaP cell growth (Figure 6K) and that in VCaP cells, AEW541 was more effective than CDSS+MDV3100 (Figure 6L).

A drug-screening experiment in the 10.5-mo LNCaP-CRPC cells, which also expressed little AR (see Figure 8A), revealed overall very similar response patterns to the 5-mo LNCaP-CRPC cells except for several noticeable differences in the group of sensitive drugs (Supplementary Figure S4). For instance, the 10.5-mo LNCaP-CRPC cells became more resistant to 10 μM ABT-199 and AEW541 although they were still sensitive to higher concentrations of both inhibitors. In addition, the 10.5-mo LNCaP-CRPC cells displayed resistance to 50 μM SB431542 and 100 μM SU 5402 (Supplementary Figure S4). These results suggest that compared to the 5-mo LNCaP cells, the 10.5-mo LNCaP-CRPC cells developed further resistance to the initially sensitive drugs.

Interestingly, LNCaP-abl cells, which were AR^+^ PSA^−^ (Figure 3B; 3), showed both similar and different responses compared to the 5-mo LNCaP-CRPC (AR^−^ PSA^−^; Figure 3B) cells and to LNCaP-GFP (AR^+^ PSA^+^; Figure 3B) cells (Figure 5) as well. For example, LNCaP-abl cells exhibited a dose-dependent sensitivity to both MDV-3100 and Bicalutamide (Figure 5A). LNCaP-abl cells also demonstrated exquisite sensitivity to docetaxel and Avicin (Figure 5A–5B). On the other hand, LNCaP-abl cells were reproducibly more resistant to 10 μM of ABT-199 (Figure 5A; data not shown). These results highlight differences in various castration-resistant LNCaP sublines such as observed here between our LNCaP-CRPC vs. LNCaP-abl cells.

### Further evidence that LNCaP-CRPC cells remain sensitive to kinase inhibitors

Prompted by the preceding observations that LNCaP-CRPC cells are sensitive to some kinase inhibitors (Figure 5D; Supplementary Figure S4), we screened 5-mo LNCaP-GFP (parental), 5-mo LNCaP-MDV, and 16-mo LNCaP-MDV cells against a collection of 752 inhibitors of >140 human kinases (Supplementary Figure S5A; Supplementary Table S3). This library contained 183 inhibitors that target multiple Ser/Thr kinases (e.g., CDKs, GSK3, MAPK/ERK/MEK), 155 inhibitors of receptor tyrosine kinases (RTKs), 88 inhibitors of PI3K/AKT/mTOR signaling, and inhibitors that target many other kinases (Supplementary Figure S5A; Supplementary Table S3). The 3 LNCaP cell types were subjected to a 3-step screening protocol, i.e., primary and secondary screens and validation (Supplementary Figure S5B-S5C) and only those inhibitors (at 10 μM) that demonstrated >40% inhibition in both primary and secondary screens were considered true ‘hit’ compounds. Primary screen resulted in 128, 31, and 30 hits whereas secondary screen resulted in 44, 11, and 10 hits in LNCaP-GFP, 5-mo LNCaP-MDV, and 16-mo LNCaP-MDV cells, respectively (Supplementary Figure S5C; Table 1). The decreased number of hits in the LNCaP-MDV cells indicates that the chronically selected CRPC cells are resistant to most kinase inhibitors tested.

**Table 1:** Secondary screen hit compounds in kinase inhibitor screening of parental, 5-mo. and 16-mo. LNCaP-CRPC cells

Pathway	Parental (44hits)	5 mo (11hits)	16 mo (10 hits)
Serine/Threonine/Cell Cycle	EMD 341251 (Cdk4/D1)	EMD 341251 (Cdk4/D1)	EMD 341251 (Cdk4/D1)
	Dinaciclib (SCH727965)(Cdk)	EMD 361550(GSK-3 Inhibitor IX)	EMD 219476(Cdk4/D1)
	EMD 217695(Cdk1)	EMD 238811(Cdk9 Inhibitor II)	
	SY-Flavopiridol (Cdk)		
	EMD 203882 (Cdk/CK1)		
	EMD 217696 (Cdk1)		
	SY-SNS-032 (Cdk)		
	EMD 400090 (CK-1)		
	Tocris 1777 (MEK)		
	Se S1527 (p38 MAPK)		
PI3K/Akt/mTOR	NVP-BGT226 (PI3K, mTOR)	NVP-BGT226 (PI3K, mTOR)	NVP-BGT226 (PI3K/mTOR)
	EMD 124011 (Akt Inhibitor IV)	EMD 124011 (Akt Inhibitor IV)	EMD 124011 (Akt Inhibitor IV)
	SY-GSK2126458 (PI3K)		
	SY-GSK690693 (Akt/PKB,Akt 1, 2, 3)		
	PF-05212384 (PI3Kα, PI3Kγ, mTOR)		
	EMD 528116 (PI3Kα Inhibitor VIII)		
	SY-GNE-493 (Pan-PI3K, pan PI3K/mTOR)		
	INK 128 (mTOR)		
	EMD 528111 (PI3Kα)		
RTK		EMD 572660 (PDGFRβ)	EMD 572660 (PDGFRβ)
	Se S1244 (c-Kit, VEGFR, PDGFR)		Se S1244 (c-Kit,VEGFR, PDGFR)
	Se S1178 (VEGFR-PDGFR)		
	SY-AG13958 (VEGFR)		
	Apatinib (YN968D1) (VEGFR)		
	BGJ398 (NVP-BGJ398) (FGFR)		
	Desmethyl Erlotinib (CP-473420) (EGFR)		
	480 (PDGFR)		
	GSK1904529A (IGF-1R and IR)		
	EMD 521233 (PDGFR)		
	Tocris 2693 (cMET, c-Met)		
	INCB28060 (c-Met)		
Multi Kinase	EMD 521275 (PDK1,Akt, Flt)	EMD 521275 (PDK1,Akt, Flt)	EMD 521275 (PDK1,Akt, Flt)
	EMD 420298 (Multi Kinase Inhibitor)		EMD 420298 (Multiple protein kinases)
	SY-A-674563 (AKT,CDK, PKA)		EMD 569397 (Multiple protein kinases)
	EMD 528100 (DNA-PK,PI3-K, and mTOR)		Enzo EI-156 (PKC, CDK1/B, CDK2/A, CDK4/D, CDK5, GSK-3, Pim-1)
	SY-PIK-75 (PI3K, p110α,DNA-PK)		
	SY-Sorafenib (B-Raf,VEGFR, Mek, Erk)		
	Se S1134 (JAK2/3, Aurora)		
	Se S1205 (PI3K/Akt/mTOR and DNA-PK)		
Aurora kinase	TAK-901 (Aurora Kinase)	CCT137690 (Aurora Kinase A, B, C)	
IKK	EMD 401482 (IKK-2 Inhibitor V)	Tocris 2539 (IKK)	
JNK	EMD 420136 (JNK Inhibitor IX)		
Miscellaneous	Tocris 2471 (Syk)	Tocris 2471 (Syk)	
	Se S1485 (PLK)	LDN193189 (TGF-beta/Smad)	
	CH5424802 (ALK)		

* Compounds shared between cell types are indicated in red.

**Figure 7:**
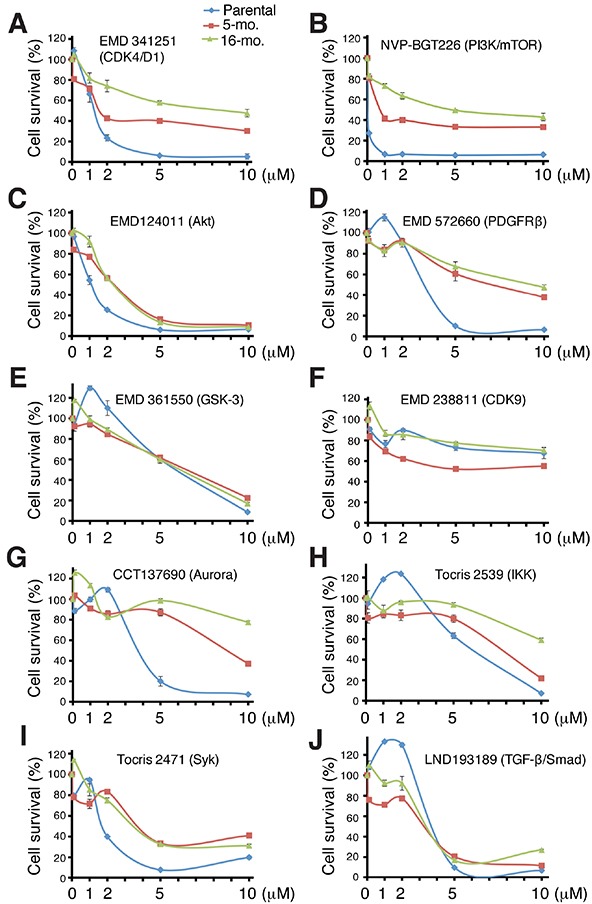
Validation studies of 10 ‘hit’ compounds in the secondary screen Three LNCaP cell types, LNCaP-GFP (parental), 5-mo and 10-mo LNCaP-MDV cells were treated with freshly purchased 10 compounds at 0, 0.1, 1, 2, 5, and 10 μM for 72 h. Live cell numbers were determined via an AlmarBlue assay. The results were presented as % cell survival relative to DMSO control. Cell survival curves were generated by applying the smooth marked scatter plot in Excel with standard deviation as error bar. For IC50 determination, we used prism to perform the nonlinear regression fit between the log concentration and the response with variable slope.

An analysis of the secondary screen hit compounds (Table 1) and validation studies (Figure 7) on most of the hits using freshly purchased compounds at a range of concentrations (i.e., 0.1, 1.0, 2.0, 5.0, and 10.0 μM) revealed some interesting results. For example, EMD341251, which blocks the CDK4/D1 activity (Supplementary Table S2), not only inhibited LNCaP-GFP cells but also, partially, LNCaP-MDV cells (IC_50_=1.15, 1.37, and 6.71 μM for parental, 5-mo and 16-mo LNCaP-MDV cells, respectively) although, not surprisingly, the 5-mo and, especially, the 16-mo LNCaP-MDV cells were more resistant (Figure 7). These results suggest that the castration-resistant LNCaP-CRPC cells partially rely on CDK4/D1 for their continued proliferative capacity, consistent with recent observations by others [4, 21]. NVP-BGT226, a dual PI3K and mTOR inhibitor, exhibited a similar inhibitory pattern in LNCaP-GFP and LNCaP-MDV cells to EMD341251 (Figure 7B; Table 1). Interestingly, EMD124011, an AKT inhibitor (Supplementary Table S2), exhibited impressive growth-inhibitory effects on both LNCaP-GFP and LNCaP-MDV cells (IC_50_=1.085, 2.417, and 2.045 μM for parental, 5-mo and 16-mo LNCaP-MDV cells, respectively; Figure 7C). Together, these observations suggest that LNCaP-MDV cells continue to require and depend on PI3K/mTOR/AKT signaling for their survival, consistent with LNCaP cells having *PTEN* mutation and with well-established involvement of the PI3K/mTOR/AKT pathway in CRPC [4, 40, 41].

Several other kinase inhibitors also displayed varying levels of inhibitory effects on LNCaP-MDV cells, which included EMD572660 (inhibiting PDGFRβ; IC_50_=2.628, 4.943, and 4.988 μM for parental, 5-mo and 16-mo LNCaP-MDV cells, respectively; Figure 7D), EMD361550 (targeting GSK-3α/α; IC_50_=6.362, 5.638, and 4.638 μM for the 3 cell types, respectively; Figure 7E), EMD238811 (inhibiting CDK9; Figure 7F. Note IC_50_ values could not be determined), CCT137690 (targeting Aurora kinases A/B/C; Figure 7G), Tocris 2539 (inhibiting IKK; IC_50_=5.04, 6.68, and 6.62 μM for parental, 5-mo and 16-mo LNCaP-MDV, respectively; Figure 7H), Tocris 2471 (inhibiting Syk; IC_50_=1.95, 2.82, and 2.08 μM for parental, 5-mo and 16-mo LNCaP-MDV cells, respectively; Figure 7I), and LND193189 (inhibiting TFG-β/Smad; IC_50_=3.12, 4.61, and 2.27 μM for parental, 5-mo and 16-mo LNCaP-MDV cells, respectively; Figure 7J). Many of these signaling pathways have been implicated in PCa progression and/or therapy resistance [42–48].

Interestingly, these latter inhibitors showed slight growth-promoting effects at 0.1-2 μM followed by inhibitory effects in the parental LNCaP-GFP cells (Figure 7D–7J). In contrast, the LNCaP-MDV cells did not manifest this bi-phasic response pattern (Figure 7D–7J). Taken together, the kinase inhibitor library screening indicates that castration-resistant LNCaP-MDV cells engage multiple kinase signaling pathways, including PI3K/AKT/mTOR, PDGFR, GSK-3, IKK, Syk, and TGFb/Smad, for their survival and proliferation.

### Evidence for cyclic changes of AR and PI3K/mTOR/AKT signaling molecules in LNCaP CRPC cells and for epigenetic involvement during MDV3100 treatment

**Figure 8:**
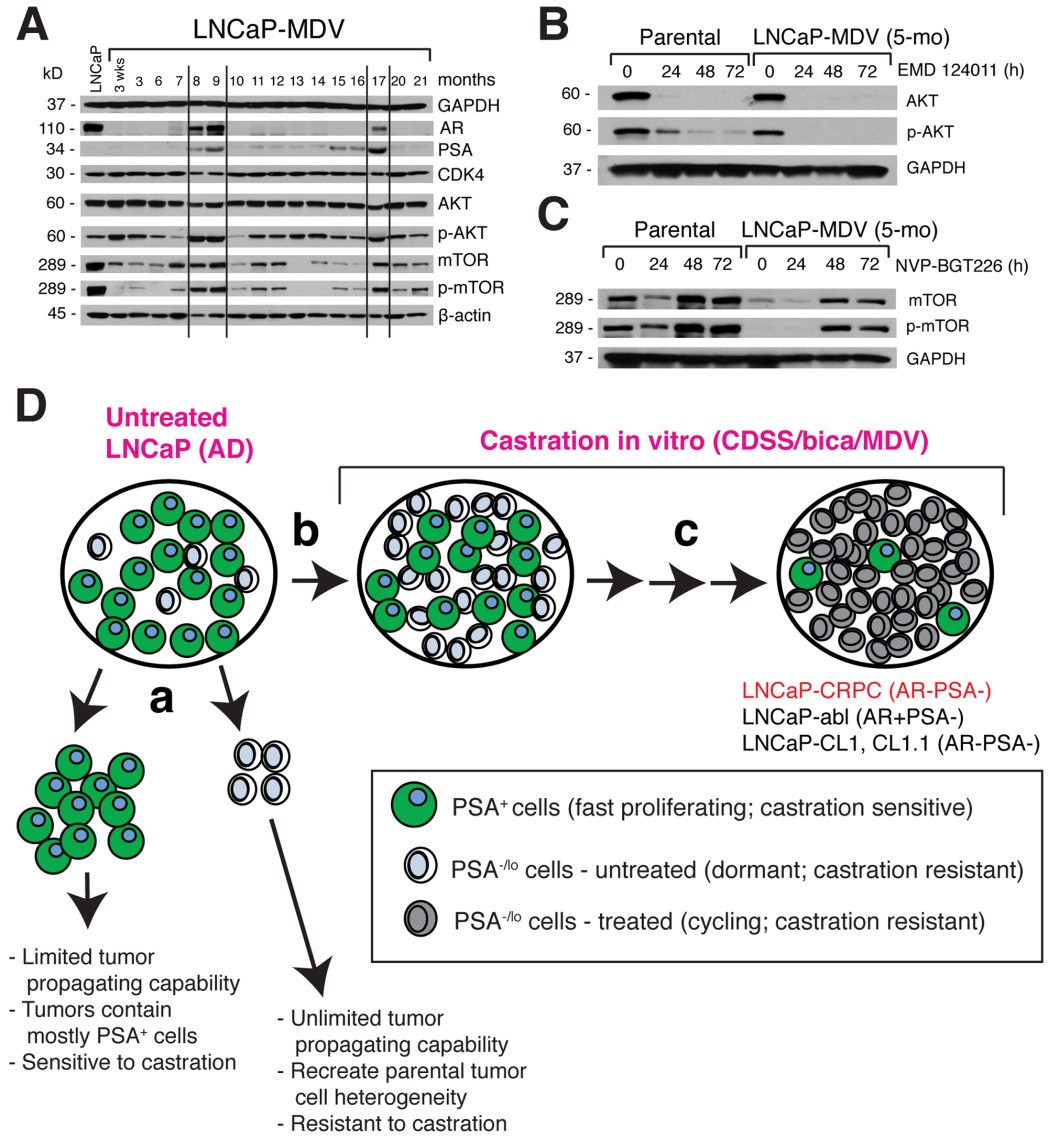
Dynamic molecular changes in AR/PSA and AKT/mTOR pathway molecules during chronic treatment of LNCaP cells with MDV3100 and a model **A.** Immunoblotting analysis of the molecules indicated in LNCaP cells treated with MDV3100 from 3 weeks to 21 months. Total cell lysates (60 mg/lane) were used and untreated LNCaP-GFP cells were employed as the control (lane 1). Both GAPDH (top) and b-actin (bottom) WB were used as loading controls. Note cyclic changes in AR, PSA, and several other molecules (demarcated by vertical lines). **B-C.** Differential response of LNCaP-GFP (parental) and 5-mo. LNCaP-MDV cells to EMD124011 (B) and NVP-BGT226 (C) treatment for the time periods indicated. **D.** A hypothetical model of how the LNCaP-CRPC sublines might have been generated in the current study. See Text for discussion. The legend is indicated below.

We further examined the long-term molecular changes in MDV3100-treated LNCaP cells (Figure 8A). As observed earlier (Figure 3B), LNCaP cells treated with MDV3100 for up to 7 months lacked expression of AR and PSA proteins (Figure 8A). Accompanying the loss of AR, cells activated AKT at 3 weeks post MDV3100 treatment as evidenced by increased p-AKT/AKT ratio, which subsequently declined (Figure 8A; Figure S6A). The LNCaP-MDV cells at 5-6 months still expressed slightly higher levels of p-AKT than LNCaP-GFP cells (Figure 8A; data not shown). When parental LNCaP-GFP cells were treated with EMD124011, AKT was lost within 24 h accompanied by a rapid decrease of p-AKT (Figure 8B). Strikingly, EMD124011 induced even faster loss of AKT and p-AKT in the 5-mo LNCaP-MDV cells (Figure 8B), potentially explaining the exquisite sensitivity of LNCaP-MDV cells to EMD124011 (Figure 7C). Surprisingly, both mTOR and p-mTOR levels significantly decreased at 3 weeks to 7 months post MDV3100 treatment (Figure 8A). Indeed, the 5-mo LNCaP-MDV cells showed low levels of mTOR and barely detectable p-mTOR (Figure 8C; time 0 lanes). Treatment of LNCaP-GFP cells with the PI3K/mTOR inhibitor NVP-BGT226 led to decreased mTOR/p-mTOR levels at 24 h but increased mTOR/p-mTOR levels at 48 and 72 h (Figure 8C). The 5-mo LNCaP-MDV cells showed overall a similar response pattern to NVP-BGT226 (Figure 8C). These results suggest that the sensitivities of LNCaP-GFP and LNCaP-MDV cells to NVP-BGT226 (Figure 7B) likely resulted from the inhibition of PI3K.

To our great surprises, AR, PSA, p-AKT, and mTOR/p-mTOR levels showed cyclic changes throughout the 21-month treatment period such that at 8-9 months, all 4 proteins increased and then declined afterwards, and increased again at ~17 months then decreased or lost (Figure 8A). This cyclic pattern of changes implicates the involvement of certain epigenetic mechanisms in MDV3100-treated LNCaP cells. In support, when we probed the same panel of time-related samples for four histone 3 (H3) marks, i.e., H3K27ace (an enhancer mark), H3K4me1 (also an enhancer mark), H3K4me3 (an activation mark), and H3K27me3 (a repressive mark), we also observed cyclic changes in these marks, in particular, H3K27me3 (Supplementary Figure S6B). In further support of epigenetic mechanisms, when we put the 14-mo AR^−^/PSA^−^ LNCaP-MDV cells (Figure 8A) back into the normal culture medium (RPMI+7% FBS), AR (but not PSA) started to re-emerge around 6 months and became strong at 8 months (Supplementary Figure S6C).

## DISCUSSION

In the present study, we have, for the first time, longitudinally tracked the response of LNCaP cells, to castration for up to ~2 years. The most important distinction of our study from all earlier related work is that we employed the PSAP-GFP/RFP lineage-tracing reporters to monitor the dynamic changes in the PSA^−/lo^ and PSA^+^ subpopulations of the LNCaP cells upon blockade of androgen signaling. One of the major findings is that 3 different castration regimens all gradually result in decrease and loss of the mRNAs and proteins of AR and its target PSA, leading to increasing numbers of PSA^−/lo^ cells. In regular serum-containing LNCaP cultures, the majority of cells are PSA^+^; however, there is a minor population of PSA^−/lo^ LNCaP cells (Figure 1B; Figure 8D) [2]. Flow cytometry analysis of castrated LNCaP cells at multiple consecutive time points indicates that castration leads to loss of PSA^+^ cells. Time lapse-based single cell tracing also provides direct evidence that the PSA^+^ LNCaP cells respond to MDV3100 by undergoing rapid cell-cycle arrest and cell death (Figure 4B). Loss of PSA^+^ LNCaP cells should lead to the enrichment of PSA^−/lo^ cells (Figure 8D, b). These observations are consistent with our recent demonstration that freshly purified PSA^+^ LNCaP cells are more sensitive to apoptosis induction by castration and chemotherapeutic drugs compared to the PSA^−/lo^ cells [3]. In ~2-3 months, castrated LNCaP cultures have largely lost both AR and PSA proteins (Figure 3B; Figure 8A) resulting in mostly PSA^−/lo^ cells (Figure 8D, c). These results are not particularly surprising considering that PSA^+^ PCa cells all express high levels of nuclear AR [2, 3] and, in the absence of androgens (CDSS) and/or with the blockade of AR signaling (CDSS+bicalutamide and MDV3100), AR will be degraded leading to the death of AR^+^/PSA^+^ PCa cells and time-related enrichment of AR^−^/PSA^−^ cells. On the other hand, there exists a possibility that some PSA^+^ LNCaP cells, under castration, may undergo de-differentiation and thus contribute to the enlarging pool of PSA^−/lo^ cells. We are currently developing genetic tracing tools that can allow us to directly test this possibility.

The resultant PSA^−/lo^ LNCaP-CRPC cells are highly resistant to further castration by high levels of MDV3100 (i.e., 200 μM) as well as to chemotherapeutic drugs (Figure 5A–5B). What mechanisms could be responsible for this multi-drug resistance (MDR) phenotype in LNCaP-CRPC cells? We have previously shown [2, 3] that the PSA^−/lo^ PCa cells can undergo asymmetric cell division, possess stem cell gene expression and epigenetic profiles, possess unlimited tumor-propagating capabilities and can recreate the tumor cell heterogeneity, and, importantly, are intrinsically resistant to castration, chemodrugs, and prooxidants (Figure 8D, a). In contrast, the PSA^+^ PCa cells only undergo symmetric cell division, possess more limited tumor-propagating activity, and are sensitive to castration and chemodrugs [2, 3] (Figure 8D, a). Therefore, the MDR phenotype of our long-term LNCaP-CRPC cells may be self-evidently explained by that fact that they are homogeneously enriched PSA^−/lo^ cells. The LNCaP-CRPC cells have also acquired some phenotypic CSC markers including CD44, α2β1, and ABCG2, all of which have been shown by us to enrich for tumor-initiating and tumor-propagating cells in different PCa models [2, 3, 28-30]. Upregulation of ABCG2 may contribute to the MDR properties of the LNCaP-CRPC cells via mediating the efflux of the drug MDV3100 or bicalutamide. Of note, our LNCaP-CRPC cells have not undergone EMT, which has been linked to castration resistance [31–33], or neuroendocrine differentiation (Rycaj et al., unpublished observations). Also, as the LNCaP-CRPC cells lose AR, *AR* mutations (e.g., 21) unlikely represent a major mechanism mediating their resistance to further castration and other treatments.

Are LNCaP-CRPC cells susceptible to any other chemical inhibitors or therapeutics? A targeted library screening reveals that they are sensitive to ABT-199 and AEW541, thus implicating BCL2 and IGF-1R as critical survival factors for the LNCaP-CRPC cells. These observations are fully consistent with our earlier comparative cDNA microarray analysis showing the preferential expression of both molecules in the PSA^−/lo^ PCa cells [2]. A medium-throughput screening against a collection of ~750 inhibitors of >140 human kinases reveals the importance of another critical cell survival signaling pathway, i.e., PI3K/AKT, in maintaining the survival of LNCaP-CRPC cells. These results make sense because one of the earliest responses we have observed in LNCaP cells exposed to castration is cell death (Figure 1H; Figure 4). ~3 weeks after MDV3100 treatment, AKT is activated (Figure 8A), presumably to extend the survival of the treated cells. The kinase inhibitor screening also identifies partial inhibitory effects of a CDK4 inhibitor on the LNCaP-CRPC cells, indicating that these cells rely on the CDK4/D1 signaling for their proliferation. Finally, the kinase inhibitor library screening implicates several other kinases, including PDGFRβ, GSK-3, IKK, Syk, and TGFb/Smad, in the survival and/or proliferation of LNCaP-CRPC cells. All of these signaling pathways have been implicated, to some degrees, in PCa cell survival and therapy resistance [42–48]. The results with the Syk inhibitor were of particular interest as this tyrosine kinase was recently shown to regulate the invasive and metastatic properties of PCa cells via positively modulating integrin α2β1 and CD44 expression [42], the two molecules also upregulated in our LNCaP-CRPC cells. Taken together, the chemical library screenings demonstrate that the long-term LNCaP-CRPC cells have developed prominent resistance to further castrations and chemotherapeutic drugs but remain sensitive, at least partially, to inhibitors of BCL-2 and several protein kinases including IGF-1R, PI3K/AKT, CDK4/D1, PDGFRβ, GSK-3, IKK, Syk, and TGF-b/Smad. It should be noted that LNCaP cells have been shown to be insensitive to TGFβ stimulation due to genetic changes in TGFB type I receptor [49]; therefore, the inhibitory effects of TGFb inhibitors could be mediated in both TGFb-dependent and independent mechanisms.

Our long-term LNCaP-CRPC cells, despite being phenotypically PSA^−/lo^, behave apparently differently from the small subpopulation of PSA^−/lo^ LNCaP cells that pre-exist in untreated bulk cultures in that they proliferate nearly as rapidly as the untreated bulk PSA^+^ cells (Figure 8D, c) whereas the preexistent subpopulation of PSA^−/lo^ LNCaP cells are largely quiescent [2, 3]. Persistent castration may likely engage/activate significant epigenetic mechanisms. This possibility is strongly supported by the striking observations that AR, PSA, AKT, and mTOR/p-mTOR proteins all undergo cyclic changes in long-term MDV3100-treated LNCaP cells. In further support, we have also observed cyclic changes in several epigenetic regulatory histone marks, in particular, the H3K27me3 mark. Moreover, we could restore AR protein expression in AR^−^/PSA^−^ LNCaP-MDV cells by simply reverting the cells back to their normal culture medium. Although the precise epigenetic mechanisms underlying the cyclic changes in AR and other proteins remain to be determined, such mechanisms may likely have contributed to seemingly quite different castration-resistant LNCaP sublines and clones reported by different groups, with some showing upregulated AR [e.g., 9, 13, 14, 16] whereas others lacking AR/PSA expression [e.g., 10-12, 15, 17-19; this study] (Figure 8D, c). This latter point can be clearly seen in the side-by-side comparison of our LNCaP-CRPC vs. LNCaP-abl cells – the AR^−^ PSA^−^ LNCaP-CRPC and AR^+^ PSA^−^ LNCaP abl cells display many differences in their responses to various targeted therapeutics (Figure 5). This is interesting as both LNCaP-CDSS cells and LNCaP-abl cells are derived by long-term exposing the parental LNCaP cells to CDSS (9; this study). It is unclear why different groups generate LNCaP (clonal or bulk) sublines that are quite different with respect to AR/PSA expression when using similar castration protocols. Recent deep sequencing data suggest that, surprisingly, LNCaP cells are ‘hypermutable’ and individual LNCaP cells manifest great differences in their exomic sequences [50, 51].

The present study demonstrates that chemical castration including MDV3100 treatment elicits not only changes in the AR signaling pathway but also multiple other molecular changes as well as epigenetic alterations, which together reprogram androgen-responsive LNCaP cells to castration- and multidrug-resistant PSA^−/lo^ cells. The availability of large numbers of PSA^−/lo^ cells should pave the way for future high throughput screening efforts to identify novel therapeutics that specifically target this population.

## MATERIALS AND METHODS

### Cells, antibodies, and compounds

LNCaP and VCaP cells were obtained from ATCC and 293FT packaging cells from Invitrogen (Carlsbad, CA) and cultured per manufacturers' instructions. LNCaP-Abl cells [9] were a gift from Dr. Helmut Klocker (University of Innsbruck, Innsbruck, Austria) and cultured in RPMI-1640 supplemented with 10% (*v*/*v*) charcoal dextran-stripped serum (CDSS), 0.1 μM sodium pyruvate, 1% GlutaMAX, and antibiotics at 37°C in 5% CO_2_. The identity of all 4 cell types was authenticated once every year at the MDACC Cell Line Authentication Service Core using the STR (Short Tandem Repeat) method. Antibodies used in this study were presented in Supplementary Table S1. The candidate library inhibitors and MDV3100 (5 month) hit compounds are presented in Supplementary Table S2. A full list of the library of kinase inhibitors is shown in Supplementary Table S3 and the secondary hit compounds are presented in Table 1.

### Generation of LNCaP-CRPC cell lines

LNCaP cells freshly infected with PSAP-GFP or the PSAP-GFP/DsRed lentiviral constructs were plated in complete RPMI media and allowed to recover for 48 h under normal culture conditions. Cells were then switched to phenol red-free RPMI containing 10% CDSS, 10% CDSS plus bicalutamide (10 μM) (CDSS+Bic), or MDV3100 (10 μM). Cells were treated continuously for up to ~2 years with drugs replenished every 48 h and intermittently used in various assays described in the text. The resultant long-term castration-resistant LNCaP sublines were designated LNCaP-CRPC. Age-matched and untreated LNCaP-GFP cells (i.e., LNCaP cells infected with the PSAP-GFP vector) were used as controls.

### Lentiviral infection of PCa cells

Basic lentiviral procedures were previously described [2] and the key vectors used in the present study are presented in Figure 1A and Supplementary Figure S1A. Lentivirus was produced in 293FT packaging cells and titers were determined using GFP positivity in HT1080 cells. LNCaP cells were infected, generally, at a multiplicity of infection (MOI) of 25.

### Cell survival, apoptosis, and cell cycle analysis

To determine total live cell numbers, 5×10^3^ LNCaP and LNCaP-CRPC cells were plated in triplicate or quadruplicate in 12-well plates and cultured for 10 days, with fresh medium fed every 3 days. At the end, viable cell numbers were counted using trypan blue exclusion assays. To determine the effect of MDV3100 on LNCaP parental cells, we performed FACS analysis using the Vybrant Apoptosis Kit (#V23200; Molecular Probes, Invitrogen) according to the manufacturer's instructions. Briefly, LNCaP cells infected with the PSAP-GFP reporter construct were treated with MDV3100 (10 μM) every 48 h for 4 weeks. At the end, cells were harvested and stained with Biotin-X annexin V followed by Alexa Fluor 504 streptavidin. Finally, cells were stained with propidium iodide (PI) and analyzed by flow cytometry using a FACS Aria II (BD Biosciences). For cell cycle (DNA content) analysis, 5×10^5^ cells were plated in a 6 well plate and allowed to grow for 24 h. Cells were harvested, washed, and resuspended in 1.5 ml of 1x phosphate buffered saline (PBS) until all cells were in suspension. Next, 3.5 ml of 200 proof ethanol was added drop-wise to the suspension while vortexing. After storing cells at 4°C for 12 h, cells were washed with 1xPBS and resuspended in PPR solution (1xPBS, 10 μg/ml PI, 20 μg/ml RNAse A, 0.5% Tween 20, and 0.5% BSA), and analyzed by flow cytometry on a FACS Aria II flow cytometer.

### Immunofluorescence (IF) microscopy

Briefly, cells were plated on glass coverslips overnight. Cells were fixed in 4% paraformaldehyde (PFA) for 10 minutes and then washed three times with 1xPBS. To block and permeabilize, cells were incubated for 60 min at room temperature in a 10% goat serum and 0.5% Triton solution for AR staining and a 10% goat serum and 0.3% Triton solution for PSA staining. After washing once, cells were then incubated with antibodies against AR or PSA plus 10% goat serum and 0.05% Triton for 60 min at room temperature. Following thorough washing (3X) with 1xPBS, the coverslips were incubated for 60 min at room temperature with AlexaFluor 594–conjugated goat anti-rabbit or anti-mouse IgG secondary antibody (Invitrogen) (1:500) plus 10% goat serum and 0.05% Triton. After washing once, cells were stained with 4,6-diamidino-2-phenylindole (DAPI, D9452; Sigma) (1:500) for 10 min at room temperature and slides mounted with ProLong® Gold anti-fade reagent (#P36931 Invitrogen). Images were taken using a Nikon Eclipse E800 Fluorescence Microscope.

### Quantitative RT-PCR (qRT-PCR), Western blotting (WB), and fluorescence-activated cell sorting (FACS)

qRT-PCR was performed using an ABI Prism 7900HT and the TaqMan system (Applied Biosystems). The primers, probes, and assay conditions for other molecules were designed by ABI with the following information: PSA (Hs03063374_m1; assay number), AR (Hs00907244_m1), b-actin (Hs99999903_ml), and GAPDH (4326317E). For data analysis, raw counts were normalized to the housekeeping gene averaged for the same time point and condition (Δ*C*t). Counts are reported as fold change relative to the untreated control (2−ΔΔ*C*t). For WB, cells were lysed in RIPA buffer (25 μM Tris-HCl (pH 7.6), 150 μM NaCl, 1% NP-40, 1% sodium deoxycholate, 0.1% SDS) supplemented with 1% proteinase inhibitors (#P2850; Sigma). Protein lysates were resolved by SDS-PAGE electrophoresis, transferred to Whatman Western polyvinylidenedifluoride (PVDF) membrane (#Z671088; Sigma), immunoblotted with primary antibodies (Supplementary Table S1) followed by appropriate secondary antibody and visualized using the ECL Plus Western Blotting Detection Kit (#RPN2132; GE Healthcare). For FACS, cells were dissociated into single-cell suspension using trypsin/EDTA and measured for GFP^+^ percentages. In some experiments, we stained the parent LNCaP and the LNCaP-CRPC cells with antibodies to α2β1, CD44, and ABCG2 (1 mg/1×10^6^ cells) for 30 min on ice followed by washing and subsequent staining with APC-conjugated goat anti-mouse IgG (1:500) for 15 min on ice. Cells were then washed 3 times and analyzed by a BD Fortessa Cell Analyzer.

### AEW541 experiments

Several sets of experiments were conducted with the AEW541 compound, which specifically inhibits the IGF-1R (IC50=150 nM). In one set, PSA^−/lo^ and PSA^+^ LNCaP cells were sorted and plated into 96-well plates at 2,000 cells/well in 100-μL medium. 12 h later, initial cell number was assessed by WST-1 (Roche). Briefly, 10 μl of WST-1 reagent was added into each well and plates were further incubated up to 4 h at 37°C. Absorbance was measured at 450 nm using a plate reader. Cells in the rest of the wells were treated with DMSO (vehicle control) or AEW541 (10 μM) and cell number was measured by WST-1 at the indicated time points.

In another set of experiments, purified PSA^+^ and PSA^−/lo^ LNCaP cells were resuspended in DMEM/F12 supplemented with B27 (17504-044, Life Science) and N2 (17502-048, Life Science) and mixed (9:4) thoroughly with methylcellulose (04100, Stem Cell Technology), and then added DMSO (ctrl) or 10 μM AEW541, and plated in 24-well ULA plates at 2,000 cells/wells. Spheres number and size were counted in ~2 weeks. To test the combinatorial effects of MDV3100 and AEW541, bulk LNCaP or VCaP cells were seeded in 96-well plate at 2,000 cells/well in 100 μl of culture medium 12 h before treatments. Then CDSS+MDV3100 (20 μM) and AEW541 (10 μM) were added, individually or in combination. After 0 (control time point), 2, 4, 6, 8, 10 and 12 days of treatment, cell number were assessed using WST-1. Finally, we analyzed apoptosis in cells treated with AEW541. PC3, Du145, VCaP or LNCaP cells infected with the PSAP-GFP reporter construct were plated in 6-well plate at 200,000 cells per plate. Cells were treated with DMSO (vehicle control) or AEW541 (10 μM). Treated cells and controls were stained using the Vybrant Apoptosis Kit (catalog #V23200; Life Science) and analyzed by FACS 24h after the treatments to assess cell apoptosis and death in the PSA^−/lo^ and PSA^+^ populations.

### Time lapse microscopy

LNCaP cells freshly infected with the PSAP-GFP reporter construct or, alternatively, infected LNCaP cells treated with MDV3100 (10 μM) acutely or chronically for 5 months, were plated on glass-bottom dishes (Grenier Bio-One), placed on the incubator stage of Nikon Biostation Timelapse system, and maintained at 37°C, 5% CO2 and > 95% humidity in RPMI medium supplemented with 10% FBS, 1 % Pen/Strep, and MDV3100 (10 μM). Phase and GFP images were collected continuously with a 20X objective lens at a 1-h interval for ~1 week. Data analysis was performed using Nikon NIS-Elements software.

### Candidate library drug screening

To determine the sensitivity of our LNCaP-CRPC cells to castration and other therapeutics, we performed a “candidate” library drug screen in parental LNCaP cells infected with PSAP-GFP, the three LNCaP-CRPC sublines at two time points (i.e., 5 and 10.5 months), and, for comparison, LNCaP-abl cells. The candidate library contained 15 drugs and/or compounds (see Text and Supplementary Table S2). Length of incubation time and number of cells plated were optimized to achieve the most accurate, reproducible response of cells to the AlamarBlue assay (Life Technologies). Briefly, cells were plated in 96-well black clear bottom plates (BD Falcon) at 7×10^3^ cells/well and allowed to recover for 48 h before commencement of drug treatments. Candidate library compounds were diluted via manufacturer's instructions and added to plates at three concentrations based on IC_50_ values. Cells were treated with DMSO (vehicle control) or compounds for 72 h, after which AlamarBlue reagent was added in an amount equal to 10% of the volume in the well (20 μl). Cultures were incubated for 4 h. The cytotoxicity was measured using spectrophotometry of fluorescence (excitation 560 nm, emission 590 nm) using a BioTek Synergy 2 plate reader (BioTek). Percent difference in reduction between treated and control cells was calculated using the following formula: [FI 590 of test agent/FI 590 of untreated control] × 100.

### Kinase Inhibitor library screening

We also screened the parental LNCaP-GFP and the LNCaP-CRPC cells against a collection of 752 inhibitors of >140 human kinases (Supplementary Table S3). Compound screening was performed at the TTDDD core facility (Targeted Therapeutic Drug Discovery Core) located at University of Texas-Austin using its custom selected kinase-focused collection. All liquid handling steps excluding AlamarBlue dye dispensing were performed using JANUS automated workstation (Perkin Elmer). LNCaP-CRPC cells were trypsinized and re-suspended in RPMI medium. The general screening protocol is as follows. Eighty eight μL of cells were plated into 96 well flat clear-bottom black plates (BD Falcon) at 7×10^3^ cells/well and incubated overnight at 37°C in a humidified atmosphere of 5% CO_2_. Compound dilution was first prepared in separate 96 well plates by diluting 1.8 mL of compounds stock solutions dissolved in 100 % DMSO (columns 2-11) or DMSO only (column 1) into 19 mL of water to achieve a final concentration of compound at 500 μM. 1.8 μL of diluted compounds and pure DMSO were added through columns 2-11 and column 1, respectively, into the assay plates. The final concentrations of compound and DMSO were 10 μM and 0.1%, respectively. Column 1 was used for a positive control (i.e., 0% activity) where the same amount of DMSO (final concentration at 0.1%, v/w) without compounds was added. Column 12 was reserved for a negative control (100% activity) and prepared right after compound addition by aspirating 44 μL of medium, followed by dispensing 44 μL of Triton X-100 (detergent). Assay plates were incubated at 37°C for 72 h, after which cell viability was determined by adding 10 μL of 10x AlamarBlue dye to assay plates using a MicroFlo Select bulk dispenser (BioTek). After incubating at 37°C for 4 h, both absorbance (570 nm) and fluorescence intensity (Ex/Em = 530/590 nm) were simultaneously measured in a Synergy H4 plate-reader (BioTek). The z factor calculated according to the equation 1 was over 0.9, demonstrating its robustness for compound screening. A total of 752 compounds were tested in triplicate. To normalize cell number variation between wells of assay plates, total fluorescence intensity of each well was divided by corresponding absorbance and this normalized fluorescence intensity was used to calculate inhibitory activity (equation 2). Compounds showing over 40% activity were identified as hits and further validated in multiple doses using the same assay. For data analysis, z factor was calculated using equation (1). Percent inhibition for the fluorescence signal was calculated using equation (2).

(1) $$z=1-3\left(\frac{{\text{SD}}_{p}+{\text{SD}}_{n}}{\mu_{p}-\mu_{n}}\right)$$

(2) $$\%I=100\left(\frac{F_{P}-F_{i}}{F_{P}-F_{N}}\right)$$

The abbreviation are as follows: z, z factor; SD_P_ = standard deviation of the signal for positive control; SD_n_ = standard deviation of the signal for negative control; %I, inhibition (in percent); F_P_, fluorescence intensity for positive control; F_N_, fluorescence intensity for negative control; F_i_, fluorescence intensity in the presence of compound.

## SUPPLEMENTARY FIGURES AND TABLES




