# Supplementary material for: Longitudinal tracking of subpopulation dynamics and molecular changes during LNCaP cell castration and identification of inhibitors that could target the PSA−/lo castration-resistant cells
Source: Oncotarget. 2016 Feb 10;7(12):14220–40. doi: 10.18632/oncotarget.7303 (PMC4924710; doi:10.18632/oncotarget.7303)
Supplement: Supplementary file 1 [file oncotarget-07-14220-s001.pdf]

## SUPPLEMENTARY FIGURES AND TABLES

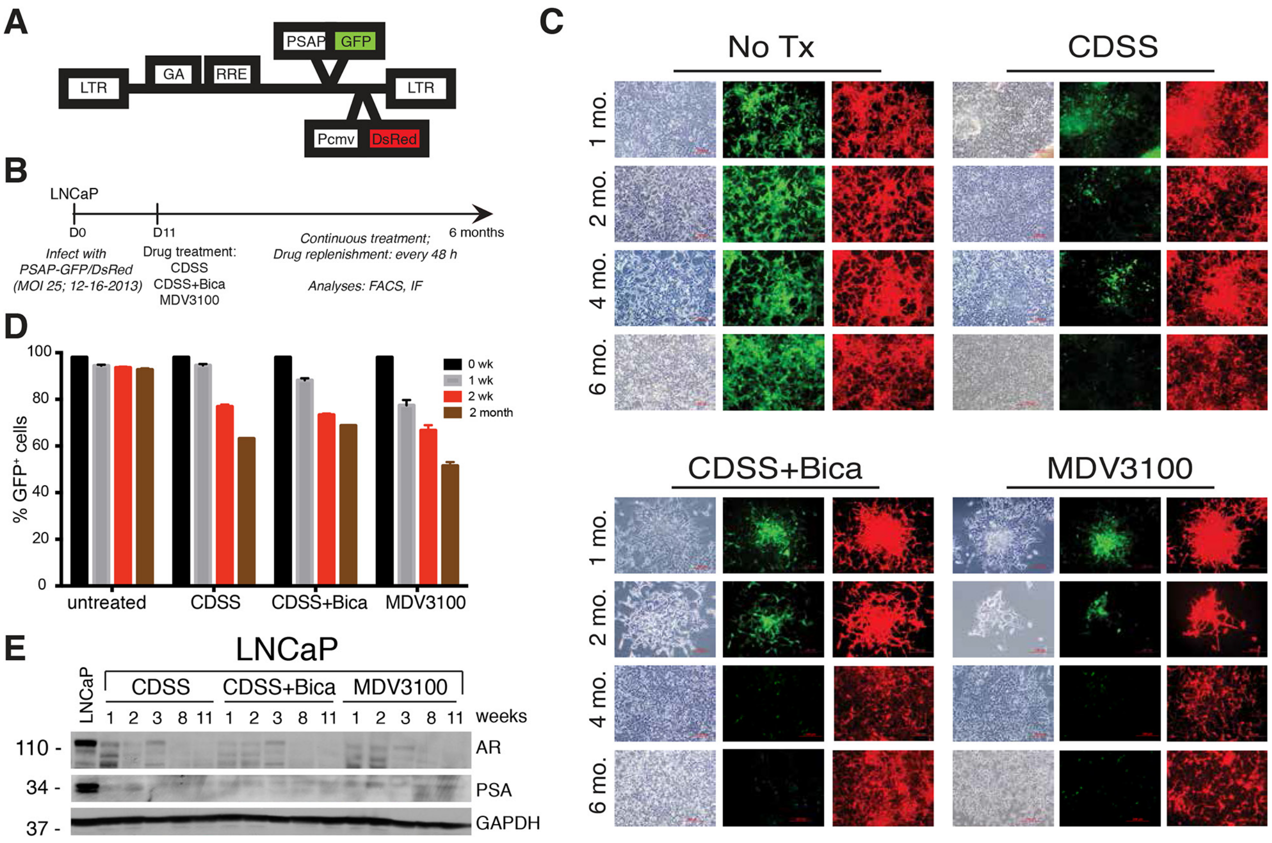


**Supplementary Figure S1: Establishment of LNCaP-CRPC cells using the PSAP-GFP/DsRed system. A.** The modified vector in which the Pcmv-DsRed cassette was incorporated. **B.** Timeline for this experiment. FACS, fluorescence-activated cell sorting; IF, immunofluorescence. **C.** Representative images of untreated (no Tx) LNCaP-GFP, CDSS, CDSS+Bicalutamide, and MDV3100 LNCaP-CRPC cells treated for the indicated intervals of time (original magnifications: 100X). Note relatively constant $RFP^+$ cells but time-dependent decrease in $GFP^+$ cells in all three castration conditions. **D.** Quantification of $GFP^+$ percentages in long-term treated LNCaP-CRPC cells. **E.** Immunoblotting of AR, PSA, and GAPDH protein expression in LNCaP-CRPC cells treated for 1, 2, 3, 8, 11 weeks in all three conditions.

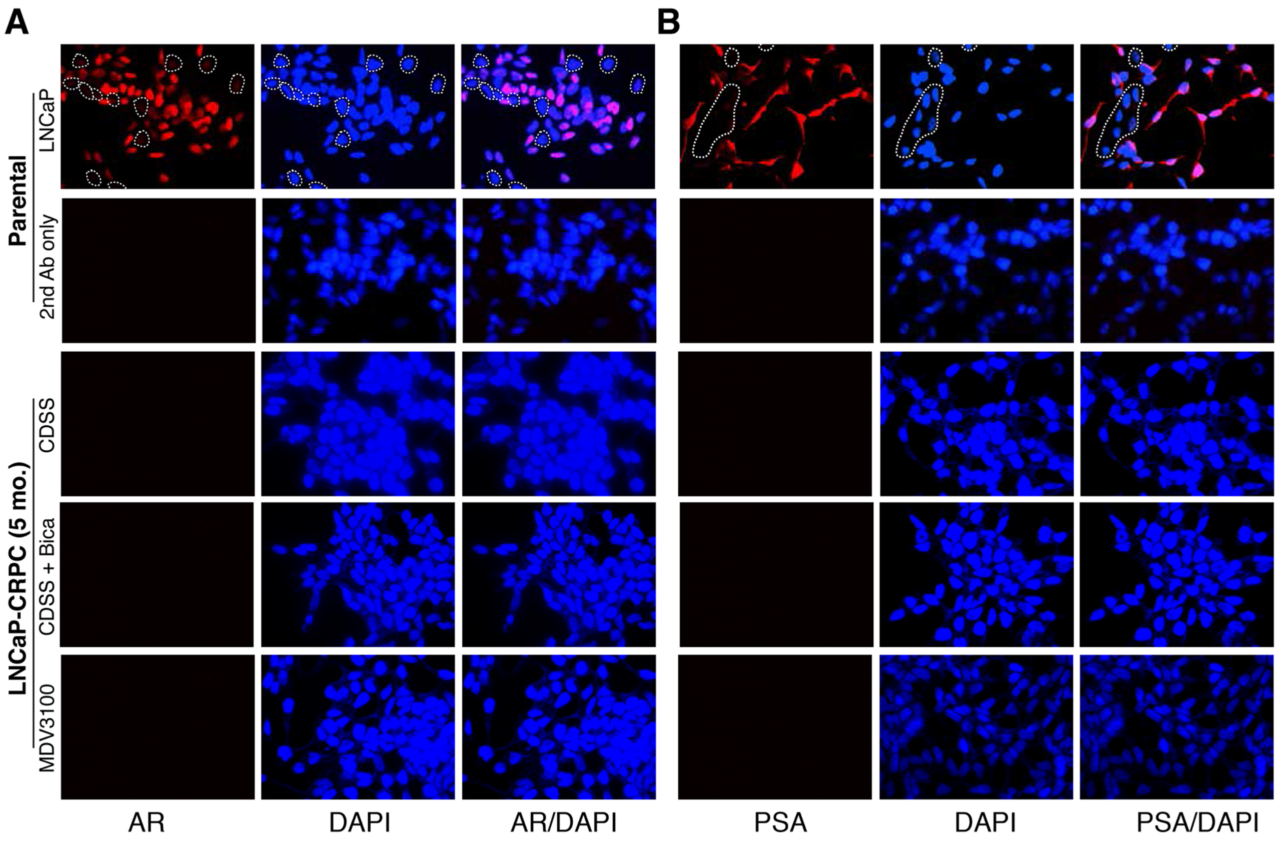


**Supplementary Figure S2: Lack of AR and PSA protein expression in 5-month LNCaP-CRPC cells.** Parental LNCaP-GFP or 5-month (5 mo.) LNCaP-CRPC cells were immunostained for AR **A.** and PSA **B.** (both at 1:10 dilution; see Supplementary Table S1 for Ab information). $AR^{-/lo}$ (A) and $PSA^{-/lo}$ (B) cells were marked by white dashed circles. Original magnifications: 400X.

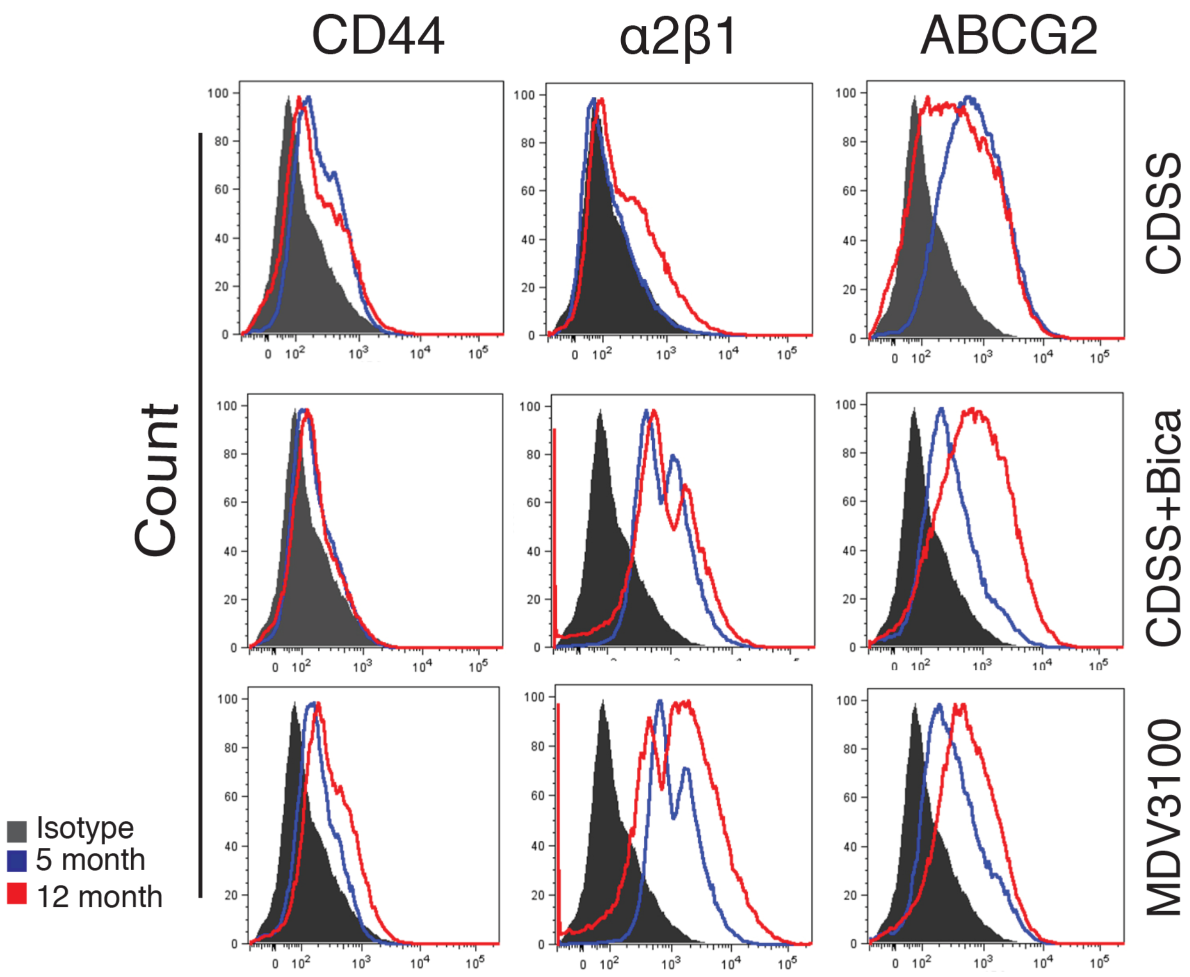


| | LNCaP | CDSS 5 | CDSS 12 | C+Bic 5 | C+Bic 12 | MDV 5 | MDV 12 |
|---|---|---|---|---|---|---|---|
| CD44 | 4.1% | 5.55% | 9.02% | 1.66% | 7.6% | 4.08% | 19.9% |
| α2β1 | 2.7% | 1.18% | 6.76% | 72.5% | 77.8% | 94% | 70.4% |
| ABCG2 | 6.9% | 49.8% | 62.1% | 26.5% | 73% | 24.5% | 67.7% |

**Supplementary Figure S3: Changes in CSC markers in LNCaP-CRPC cells.** FACS analysis of CD44, α2β1, and ABCG2 cell surface expression. Shown above are representative histogram plots in 3 LNCaP-CRPC cells treated for 5 (blue) or 12 (red) months and stained for the Abs indicated. An isotype Ab was used as a control (solid black). The mean percentages of marker-positive cells are shown in the table below. C+Bic, LNCaP cells treated with CDSS+Bicalutamide; MDV, LNCaP cells chronically treated with MDV3100.

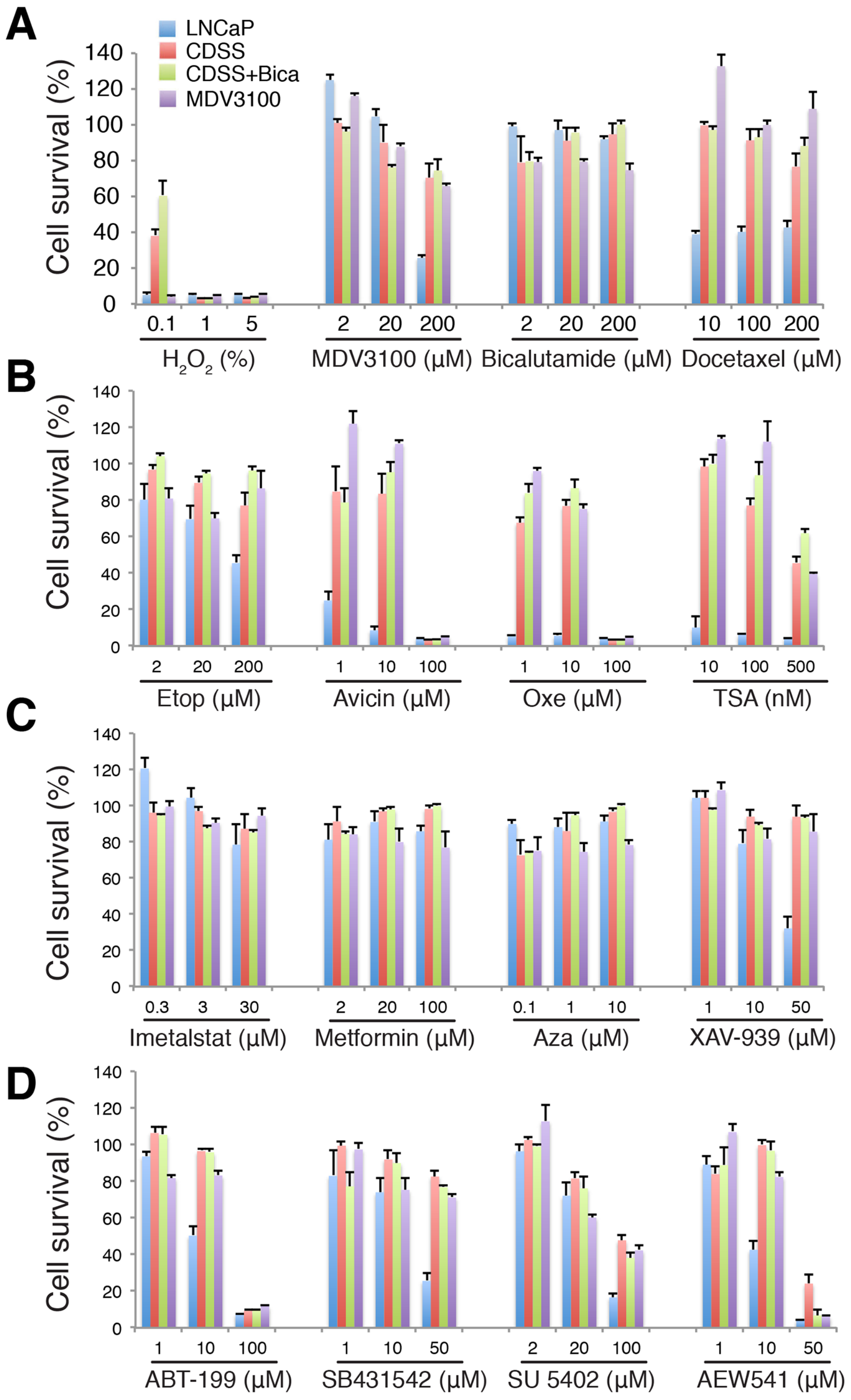


**Supplementary Figure S4: Drug sensitivities in 10.5-mo LNCaP-CRPC cells.** Presented is the relative cell survival (%), as determined by AlmarBlue assay, in LNCaP-GFP (LNCaP; 10.5-mo) and 3 types of LNCaP-CRPC (10.5-mo) cells when exposed to 15 compounds in the candidate library for 72 h. See text for more details.

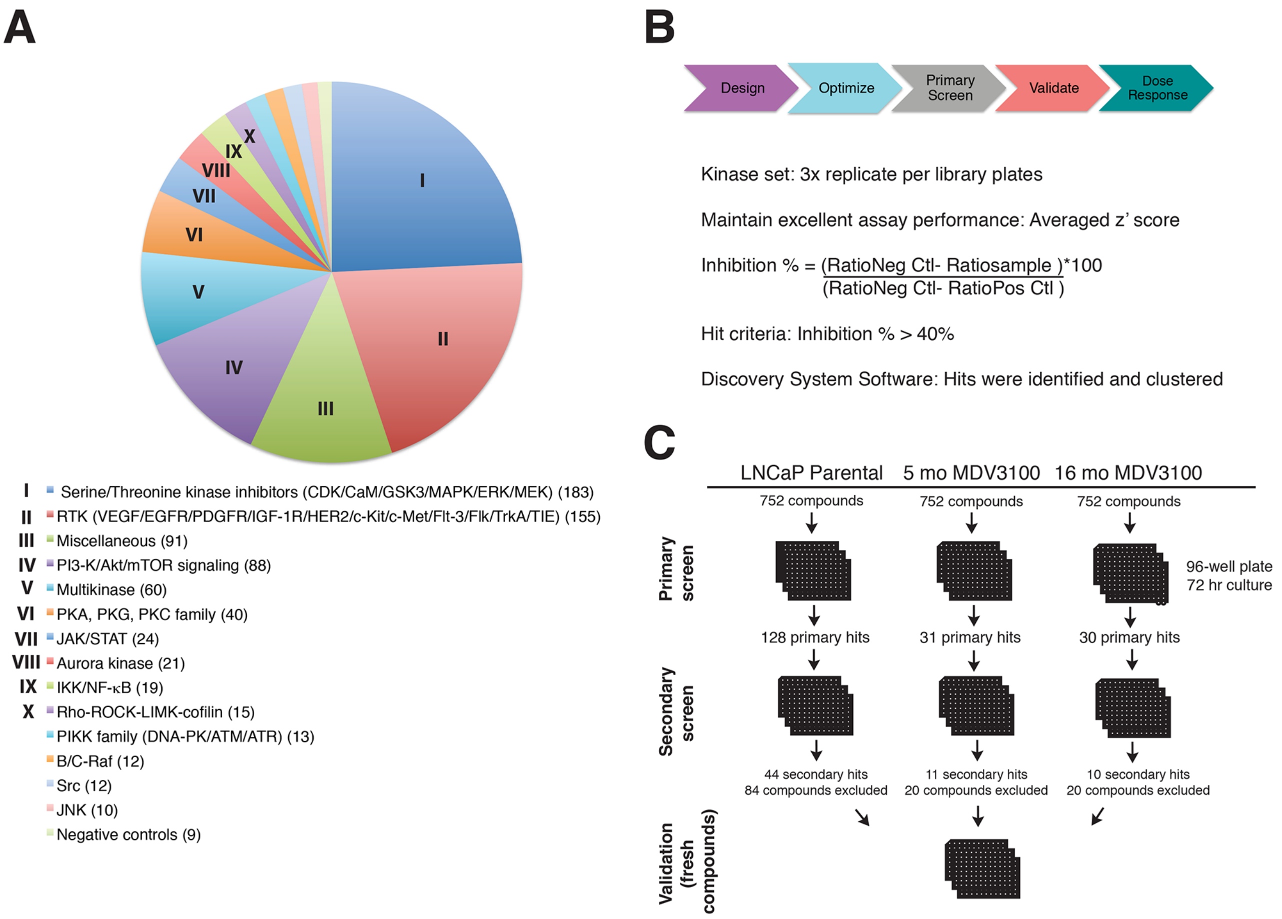


**Supplementary Figure S5: Kinase inhibitor library and drug screen workflow. A.** Pie chart presentation of the TTDDD kinase inhibitor set composed of 752 compounds that target >140 human kinases (see Methods). Kinases were grouped into major signaling pathways. Kinases targeting multiple signaling pathways were labeled as "Multikinase". Signaling pathways with ≤10 inhibitors were listed under "Miscellaneous". **B.** Drug screen workflow and critical drug screen parameters (also see Methods). **C.** Schematic depicting the primary and secondary screens and final validation in parental LNCaP-GFP, 5-mo. LNCaP-MDV and 16-mo. LNCaP-MDV cells. Total compound hits for each cell type in each screen are shown.

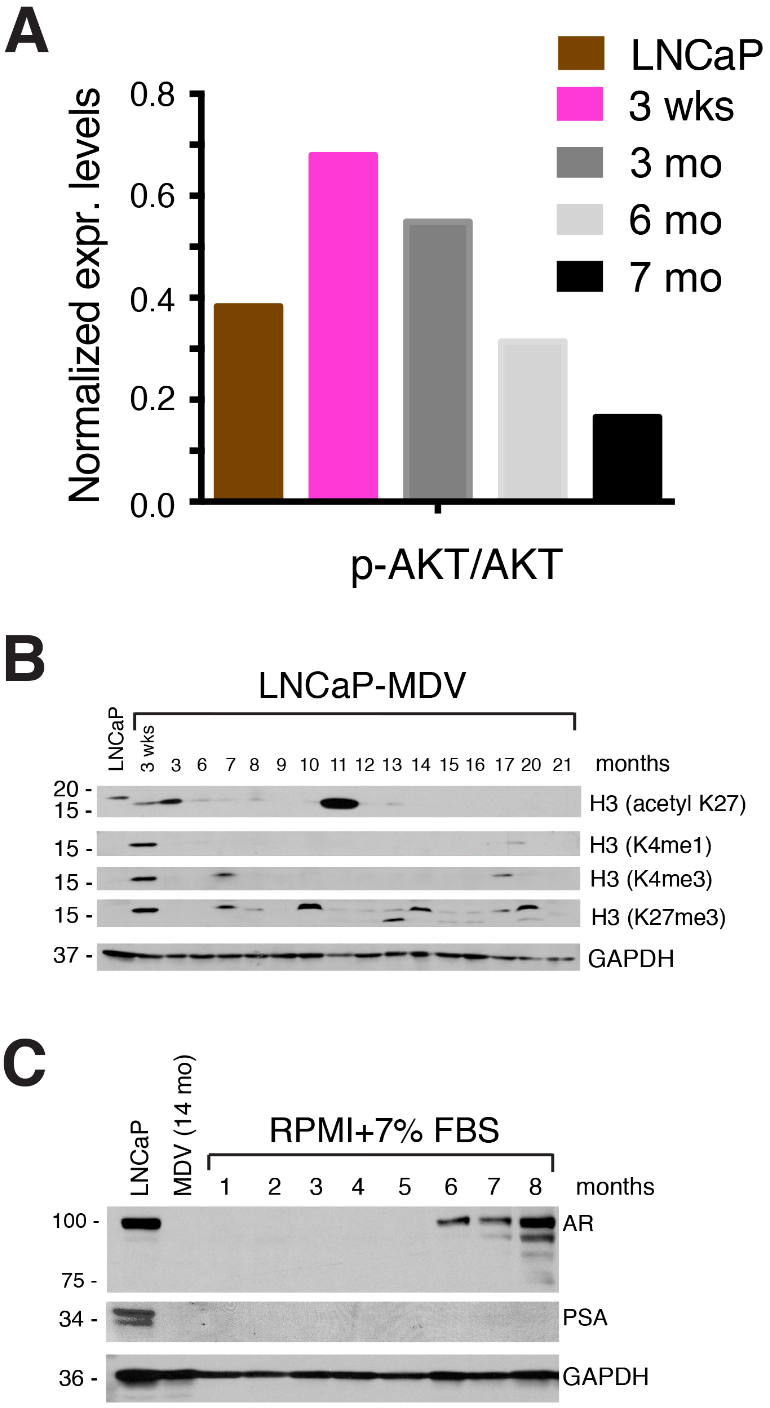


**Supplementary Figure S6: Transient activation of AKT and evidence for epigenetic mechanisms during MDV3100 treatment. A.** Early activation of AKT during MDV3100 treatment. The AKT and p-AKT (Tyr1086; Supplementary Table S1) levels detected on Western blotting during MDV3100 treatment for up to 7 months (Figure 7A; the first 5 lanes) were measured by densitometric scanning of the bands and then normalized to the respective GAPDH levels. AKT activation was then presented as the relative values of normalized p-AKT/AKT values. **B.** LNCaP-GFP (LNCaP) or LNCaP-MDV cells at different treatment time points (see Figure 7A) were used in Western blotting analysis of 4 histone 3 (H3) modification marks. Note recurrent and cyclic changes in H3K27me3. **C.** Reversion experiments. The 14-mo LNCaP-MDV cells, which were AR-negative, were put back to normal culture medium containing RPMI-7% FBS for 1-8 months. The lysates were then used in Western blot analysis for AR and PSA. Parental LNCaP-GFP (lane 1) cells were used as control.

**Supplementary Table S1: Antibodies used in the current study**

| Antibody | Source | Catalog # | Company | Remarks |
|---|---|---|---|---|
| α2β1 | Mouse | MAB2141Z | EMD Millipore | Clone BMA2.1 |
| β-actin | Mouse | 69100 | MP Biochemicals | Clone C4 |
| ABCG2 | Rabbit | AV43649 | Sigma | (C67E7) |
| ACPP | Rabbit | 15840-1-AP | Proteintech | Clone 441 |
| AKT | Rabbit | 4691 | Cell Signaling | Clone 7 |
| AR | Mouse | sc-7305 | Santa Cruz | (D9G3E) |
| AR-V7 | Mouse | AG10008 | Precision Antibody | Clone H-108 |
| BCL-2 | Mouse | 610538 | BD Transduction Labs | (D38B1) |
| CD44 | Rabbit | ab51037 | Abcam | Clone FL-335 |
| CDK4 | Rabbit | 12790 | Cell Signaling | (7C10) |
| E-cadherin | Rabbit | sc-7870 | Santa Cruz | Clone 32/N |
| EGFR | Rabbit | 4267 | Cell Signaling | (Y92) |
| FKBP5 | Rabbit | 8245 | Cell Signaling | (Ser473) (D9E) |
| GAPDH | Rabbit | sc-25778 | Santa Cruz | (Tyr1086) |
| H3 | Rabbit | 39163 | Active Motif | (Ser2448) (D9C2) |
| H3 (K27acetyl) | Rabbit | ab4729 | Abcam | (Tyr751) (88H8) |
| H3 (K27me3) | Rabbit | ab8895 | Abcam | (Ser240/244) |
| H3 (K4me3) | Rabbit | 07-473 | Millipore | (D68F8) |
| H3 (K4me1) | Rabbit | 07-436 | Millipore | Clone C-19 |
| IGF-1Rβ | Rabbit | 3027 | Cell Signaling | Clone C-20 |
| mTOR | Rabbit | 2983 | Cell Signaling | (54D2) |
| N-cadherin | Mouse | 610921 | BD Transduction Labs | Clone S-20 |
| PDGFβ | Rabbit | ab32570 | Abcam | Clone BMA2.1 |
| phospho-AKT | Rabbit | 4060 | Cell Signaling | Clone C4 |
| phospho-EGFR | Rabbit | 2220 | Cell Signaling | (C67E7) |
| phospho-mTOR | Mouse | 5536 | Cell Signaling | Clone 441 |
| phospho-PDGFβ | Rabbit | 3166 | Cell Signaling | Clone 7 |
| phospho-S6 | Rabbit | 5364 | Cell Signaling | (D9G3E) |
| PSA | Goat | sc-7638 | Santa Cruz | Clone H-108 |
| PSMA | Mouse | DM1037 | Acris Antibodies | (D38B1) |
| SOX-9 | Goat | sc17341 | Santa Cruz | Clone FL-335 |
| S6 | Mouse | 2317 | Cell Signaling | (7C10) |
| Vimentin | Goat | sc-7558 | Santa Cruz | Clone 32/N |

**Supplementary Table S2: Candidate library inhibitors (top) and LNCaP-MDV (5 months) (bottom) hit compounds**

| Inhibitor | Target | IC50 | Supp Ref |
|---|---|---|---|
| MDV3100 | AR | 36 nM (LNCaP) | |
| Bicalutamide | AR | 0.16 µM (LNCaP) | |
| Docetaxel | Depolymerisation of microtubules | 0.3 nM (HeLa cells) | |
| Etoposide | DNA synthesis via topoisomerase II | 5.7 nM (HeLa cells) | |
| Avicin | Dephosphorylates Stat 3 | 0.3 µM (Jurkat) | 1 |
| Oxetane | Dephosphorylates Stat 3 | 0.04 µM (Jurkat) | |
| GRN163L (Imetelstat) | Telomerase | 0.1 to 1 µmol/L (CSCs in PANC1, MDA-MB231, MCF7) | 2 |
| Metformin | AMP-activated protein kinase activator | | |
| ABT-199 | BCL-2 | <0.01 nM (Ki) (RS4;11) | 3 |
| 5-Aza2'-Deoxycytidine | DNA methyltransferase | 5.600 µM (PC3); 2.822 µM (DU145) | 4 |
| Trichostatin A | HDAC | ~1.8 nM (breast cancer cell lines) | |
| XAV-939 | Wnt/β-catenin-mediated transcription via TNKS1/2 inhibition | 11 nM/4 nM (TNKS1/2) (WTK1) | 5 |
| SB431542 | TGFβR1, activin receptor-like kinase ALK5, 4 and 7 | 94 nM (ALK5) (A498) | 6 |
| SU 5402 | VEGFR and FGFR | 0.02, 0.03, 0.51 and > 100 µM for VEGFR2, FGFR1, PDGFRβ, EGFR (NIH 3T3) | 7 |
| AEW541 | IGF-1R/InsR | 150 nM/140 nM | 8 |
| | | | 9 |
| NVP-BGT226 | PI3K/mTOR inhibitor for PI3Kα/β/γ | 4 nM, 63 nM, 38 nM | |
| EMD 124011 | Akt Inhibitor IV | < 1.25 µM (786-O) | |
| EMD 341251 | Cdk4/D1 | 0.35 µM (L-1210) | |
| EMD 572660 | Tyrosine kinase receptor and angiogenic inhibitor | 3, 27, 170, ~10-500 nM for PDGFRβ, VEGFR2, FGFR1, Kit family | |
| Tocris 2471 | Syk | 10 µM (RBL-2H3) | |
| CCT137690 | Aurora Kinase A, B, C | 15 nM, 25 nM and 19 nM (range of cancer cell lines) | 10 |
| Tocris 2539 | IKK | 40, 70 and 200 nM (IKKβ, IKK complex and IKKα) | 11 |
| LDN193189 | BMP type I receptors ALK2 and ALK3 | 5 nM and 30 nM | |
| EMD 361550 | GSK-3α/β Inhibitor IX | 5 nM | |
| EMD 238811 | Cdk9 Inhibitor II | 350 nM | |

1. Haridas V, Nishimura G, Xu ZX, Connolly F, Hanausek M, Walaszek Z, Zoltaszek R, Gutterman JU. Avicin D: A Protein reactive plant isoprenoid dephosphorylates Stat 3 by regulating both kinase and phosphatase activities. PLoS One 2009;4(5):e5578.
2. Joseph I, Tressler R, Bassett E, Harley C, Buseman CM, Pattamatta P, et al. The telomerase inhibitor imetelstat depletes cancer stem cells in breast and pancreatic cancer cell lines. Cancer Res. 2010;70:9494-504.

3. Souers AJ, Leverson JD, Boghaert ER, Ackler SL, Catron ND, Chen J, et al. ABT-199, a potent and selective BCL-2 inhibitor, achieves antitumor activity while sparing platelets. Nat Med 2013;19:202-8.
4. Qin T, Jelinek J, Si J, Shu J, Issa JP. Mechanisms of resistance to 5-aza-2′-deoxycytidine in human cancer cell lines. Blood. 2009;113:659-67.
Dregalla RC, Zhou J, Idate RR, Battaglia CL, Liber HL, Bailey SM. Regulatory roles of tankyrase 1 at telomeres and in DNA repair: suppression of T-SCE and stabilization of DNA-PKcs. Aging. 2010;2: 691-708.
6. Callahan JF, Burgess JL, Fornwald JA, Gaster LM, Harling JD, Harrington FP, et al. Identification of novel inhibitors of the transforming growth factor beta1 (TGF-beta1) type 1 receptor (ALK5). J. Med. Chem. 2002;45:999-1001.
7. Sun L, Tran N, Liang C, Tang F, Rice A, Schreck R, et al. Design, synthesis, and evaluations of substituted 3-[(3- or 4-carboxyethylpyrrol-2-yl)methylidenyl]indolin-2-ones as inhibitors of VEGF, FGF, and PDGF receptor tyrosine kinases. J. Med. Chem. 1999;42:5120-30.
8. Garcia-Echeverria C, Pearson MA, Marti A, Meyer T, Mestan J, Zimmermann J, et al. *In vivo* antitumor activity of NVP-AEW541-A novel, potent, and selective inhibitor of the IGF-IR kinase. Cancer Cell 2004;5:231-9.
9. Markman B, Tabernero J, Krop I, Shapiro GI, Siu L, Chen LC, et al. Phase I safety, pharmacokinetic, and pharmacodynamic study of the oral phosphatidylinositol-3-kinase and mTOR inhibitor BGT226 in patients with advanced solid tumors. Annal Oncol. 2012;23:2399-408.
10. Bavetsias V, Large JM, Sun C, Bouloc N, Kosmopoulou M, Matteucci M, et al. Imidazo[4,5-b]pyridine derivatives as inhibitors of Aurora kinases: lead optimization studies toward the identification of an orally bioavailable preclinical development candidate. J. Med. Chem. 2010;53:5213-28.
11. Waelchli R, Bollbuck B, Bruns C, Buhl T, Eder J, Feifel R, et al. Design and preparation of 2-benzamido-pyrimidines as inhibitors of IKK. Bioorganic Med Chem Lett. 2006;16:108-12.

**Supplementary Table S3: Full Kinase Inhibitor List**

See Supplementary File 1
